# A Reversible Histone H3 Acetylation Cooperates with Mismatch Repair and Replicative Polymerases in Maintaining Genome Stability

**DOI:** 10.1371/journal.pgen.1003899

**Published:** 2013-10-24

**Authors:** Lyudmila Y. Kadyrova, Tony M. Mertz, Yu Zhang, Matthew R. Northam, Ziwei Sheng, Kirill S. Lobachev, Polina V. Shcherbakova, Farid A. Kadyrov

**Affiliations:** 1 Department of Biochemistry and Molecular Biology, Southern Illinois University School of Medicine, Carbondale, Illinois, United States of America; 2 Eppley Institute for Research in Cancer and Allied Diseases, University of Nebraska Medical Center, Omaha, Nebraska, United States of America; 3 School of Biology and Institute for Bioengineering and Bioscience, Georgia Institute of Technology, Atlanta, Georgia, United States of America; Duke University, United States of America

## Abstract

Mutations are a major driving force of evolution and genetic disease. In eukaryotes, mutations are produced in the chromatin environment, but the impact of chromatin on mutagenesis is poorly understood. Previous studies have determined that in yeast *Saccharomyces cerevisiae*, Rtt109-dependent acetylation of histone H3 on K56 is an abundant modification that is introduced in chromatin in S phase and removed by Hst3 and Hst4 in G2/M. We show here that the chromatin deacetylation on histone H3 K56 by Hst3 and Hst4 is required for the suppression of spontaneous gross chromosomal rearrangements, base substitutions, 1-bp insertions/deletions, and complex mutations. The rate of base substitutions in *hst3*Δ *hst4*Δ is similar to that in isogenic mismatch repair-deficient *msh2*Δ mutant. We also provide evidence that H3 K56 acetylation by Rtt109 is important for safeguarding DNA from small insertions/deletions and complex mutations. Furthermore, we reveal that both the deacetylation and acetylation on histone H3 K56 are involved in mutation avoidance mechanisms that cooperate with mismatch repair and the proofreading activities of replicative DNA polymerases in suppressing spontaneous mutagenesis. Our results suggest that cyclic acetylation and deacetylation of chromatin contribute to replication fidelity and play important roles in the protection of nuclear DNA from diverse spontaneous mutations.

## Author Summary

Mutations strongly predispose humans to cancer and many other diseases. Despite significant progress, we still do not fully understand the molecular mechanisms that protect us from mutations. Human DNA is part of a highly organized complex called chromatin. Chromatin regulates our development, metabolism, and behavior. Special enzymes modify chromatin by the addition and removal of chemical groups. Acetylation and deacetylation of chromatin have been conserved during evolution. The involvement of chromatin and its modifications in the protection of DNA from mutations is poorly understood. The yeast *Saccharomyces cerevisiae* is an excellent model for studying the connection between chromatin modifications and mutations. Using this model, we found that the deacetylation and acetylation of chromatin on histone H3 lysine 56 are required for preventing a wide range of spontaneous mutations. Future studies will determine whether acetylation and deacetylation of chromatin are involved in protecting DNA from mutations in human cells.

## Introduction

Mutations are the prerequisites for evolution and the humoral immune response. However, mutations are often detrimental due to their ability to trigger both inherited and sporadic diseases. Base substitutions, 1-bp deletions, and 1-bp insertions are the most common mutations [1], [2]. Cells can also acquire gross chromosomal rearrangements (GCRs) [3], [4], complex mutations [5], and other genetic alterations [1], [2], [6]. Though GCRs are relatively rare mutational events, they profoundly reshape genetic information. Mutations arise as a result of replication errors, defects in DNA repair, spontaneous and induced DNA damage, and several error-prone processes including somatic hypermutagenesis, mitotic gene conversion, and break-induced replication [2], [6]–[12]. DNA replication errors produce a large fraction of spontaneous mutations [7].

The bulk of nuclear DNA is replicated by the leading-strand polymerase ε and lagging-strand polymerase δ that both possess intrinsic 3′-5′ exonucleolytic activities [13], [14]. The suppression of DNA replication errors is in part achieved by the nucleotide selectivity at the active sites of replicative polymerases that permits DNA synthesis with an error rate of 10^−4^–10^−5^ [6]. The excision of incorrectly incorporated dNMPs by the 3′-5′ exonucleolytic activity of replicative polymerases further decreases the error rate ∼100-fold. In addition, mismatch repair (MMR) promotes high-fidelity DNA replication by correcting replication errors which escaped the proofreading activities of replicative polymerases. MMR is a multifunctional process, but correction of DNA replication errors is its primary function [2], [11], [15]–[21]. Eukaryotic MMR is initiated by the binding of MutSα (MSH2-MSH6 heterodimer) or MutSβ (MSH2-MSH3 heterodimer) to a mispair. After detecting a mismatch, MutSα or MutSβ activates the endonuclease activity of MutLα (MLH1-PMS2 in humans and Mlh1-Pms1 in *S. cerevisiae*) in the presence of ATP, a strand break, and PCNA loaded by RFC [22]–[25]. A MutLα incision 5′ to the mismatch initiates the downstream events leading to the correction of the mismatch [26], [27]. MMR improves fidelity of DNA replication 10–10^4^-fold depending on the sequence context. Thus, replicative polymerases and MMR are the major factors in high-fidelity DNA replication [28]–[31].

Several reversible histone modifications have been implicated in DNA replication, repair, and damage response [32], [33]. Histone H3 K56 acetylation is one such modification located in the αN-helix that is adjacent to the histone fold domain [34], [35]. When histone H3 acetylated on K56 (H3K56ac) is part of a nucleosome, the acetylation is near the entry and exit sites of DNA and appears to loosen the histone-DNA contacts [34]. Nearly all newly synthesized yeast H3 histones are acetylated on K56 [36] by the histone acetyltransferase Rtt109 and histone chaperone Asf1 in S phase [37]–[40]. The loss of yeast H3K56ac enhances the sensitivity of cells to several DNA damaging drugs [35], [40]–[42] and destabilizes stalled replication forks [43]. During DNA damage response, yeast H3K56ac is required for both restoration of chromatin on repaired DNA and subsequent recovery of the cells from the DNA damage checkpoint [41]. H3K56ac has been identified in human cells where it is also involved in DNA damage response [44], [45].

The NAD-dependent histone deacetylases Hst3 and Hst4 erase H3K56ac marks from the newly generated chromatin in G2/M [36], [46], [47]. Like H3 K56 acetylation, H3 K56 deacetylation by Hst3 and Hst4 is important for DNA damage response. In the presence of DNA damage in G2/M in wild-type strains, H3 K56 deacetylation is delayed to allow DNA repair to take place [35]. Furthermore, it is known that H3 K56 acetylation and deacetylation are critical for selecting sister chromatid as the template for repair of replication-born double strand breaks by homologous recombination (HR) [48]. About 92% of chromatin histone H3 molecules are continuously acetylated on K56 residues in *hst3*Δ *hst4*Δ strains [36]. Strains lacking both *HST3* and *HST4* display spontaneous DNA damage, a strong sensitivity to genotoxic agents, a five-fold increase in mitotic homologous recombination, and an elevated level of chromosome loss in mitosis [36], [47], [49], [50]. Hst3 and Hst4 are members of the conserved sirtuin family also containing Hst1 and Hst2 [49], [51]. The targets of Hst1 and Hst2 enzymes are not well defined. A recent study reported that Hst1 is important for histone H3 K4 deacetylation in euchromatin [52].

Nuclear DNA is part of chromatin, but little is known about the relationship between chromatin and mutation avoidance. Previous studies have demonstrated that the yeast chromatin factors Caf1, Asf1, Hst3, and Rtt109 are involved in the suppression of GCRs [39], [53]–[55]. Furthermore, human CAF-1 has been shown to interact functionally and physically with the mismatch recognition factor MutSα and modulate MMR in cell-free extracts and reconstituted systems [56], [57]. A recent report has described that a depletion of the histone methyltransferase SETD2 triggers microsattelite instability and an increased mutation frequency at *HPRT* [58]. Because microsattelite instability is a hallmark of MMR defects and the MSH6 subunit of MutSα recognizes H3K36me3, these findings suggest that SET2D-dependent H3K36me3 is required for the action of human MMR *in vivo* [58].

In this work, we analyzed the impacts of both H3 K56 deacetylation and acetylation on spontaneous mutagenesis in *S. cerevisiae*. We found that H3 K56 deacetylation by the combined action of Hst3 and Hst4 plays a major role in the defense against GCRs, base substitutions, 1-bp insertions/deletions, and complex mutations. Our analysis also showed that in addition to being part of the protection from GCRs [54], H3 K56 acetylation is involved in the prevention of small insertions/deletions and complex mutations. Furthermore, our results revealed that both the acetylation and deacetylation of H3 K56 are important for genetic stabilization mechanisms that act in concert with MMR and the proofreading activities of replicative DNA polymerases to suppress spontaneous mutagenesis.

## Results

### Spontaneous mutagenesis in strains deficient in H3 K56 deacetylation

**Figure 1:**
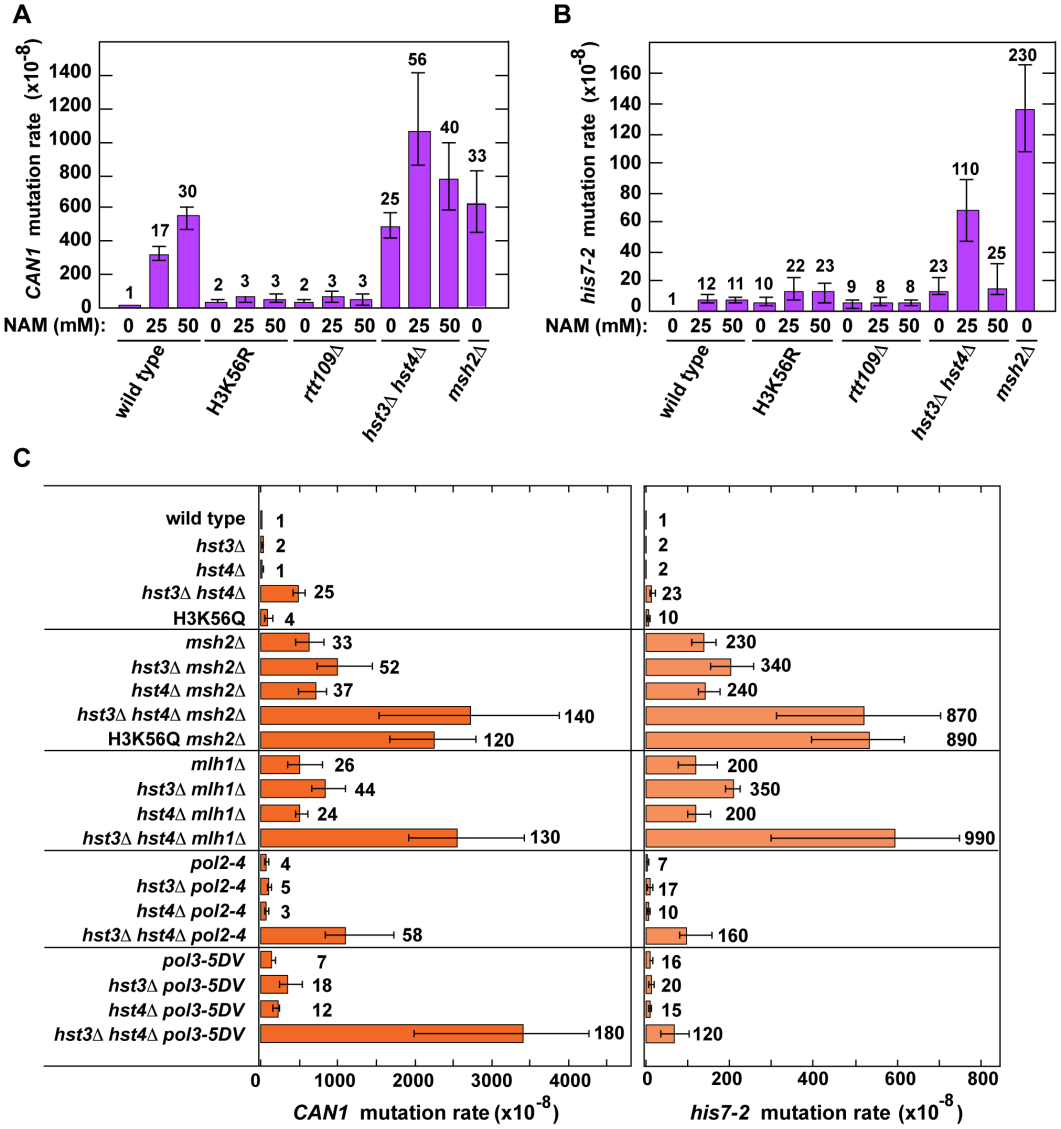
Involvement of H3 K56 deacetylation in the suppression of spontaneous mutagenesis in the yeast *S. cerevisiae*. Spontaneous mutation rates were measured as described in Materials and Methods. The data are shown as medians with 95% confidence intervals. The numbers above the bars are the relative mutation rates. (**A**) and (**B**) Effect of nicotinamide (NAM) on *CAN1* (**A**) and *his7-2* (**B**) mutation rates. The rates were measured in the haploid E134 strain (wild type) and indicated mutant derivatives exposed to 0-mM, 25-mM, or 50-mM NAM. (**C**) Effect of combining *hst3*Δ *hst4*Δ with *msh2*Δ, *mlh1*Δ, *pol2-4*, or *pol3-5DV* on spontaneous mutagenesis of *CAN1* and *his7-2*.

We started this work to investigate whether chromatin is involved in the defense against spontaneous point mutations in the haploid yeast *S. cerevisiae*. Many of our experiments relied on *CAN1* and *his7-2* reporters for scoring mutations. *CAN1* is a counter-selectable marker that allows the selection of any mutation that inactivates the gene including base substitutions, small insertions/deletions and complex mutations. In addition, *CAN1* can be inactivated by GCRs involving the 43-kb *CAN1-*containing region of chromosome V [3]. The *his7-2* reporter permits the selection of net +1 frameshift mutations causing a reversion to *HIS7* [59]. As shown in **Table 1**, analysis of several histone deacetylase and acetyltransferase mutants revealed that the *CAN1* and *his7-2* mutation rates for three different *hst3*Δ *hst4*Δ strains are about 25 times as high as those for isogenic wild-type strains. However, deletion of *HST3* or *HST4* alone causes little or no mutator phenotype (**Table 1**). We also established that the *CAN1* mutation rates in *hst3*Δ *hst4*Δ are very similar to those in the MMR-deficient *msh2*Δ and *mlh1*Δ strains (**Figure 1C**). Collectively, these data demonstrated that the loss of *HST3* and *HST4* strongly promotes spontaneous mutagenesis.

**Table 1:** Spontaneous mutagenesis in strains deficient in histone H3 K56 deacetylation.

	Mutation rate			
	*CAN1*		*his7-2*	
Genotype	Absolute rate (×10^−8^)	Relative rate	Absolute rate (×10^−8^)	Relative rate
E35 (wild type)	27 (23–49)	1	0.6 (0.4–1.0)	1
E35 *hst3*Δ *hst4*Δ	690 (470–850)	25	16 (8–32)	27
BY4742 (wild type)	17 (14–25)	1	-	-
BY4742 *hst3*Δ *hst4*Δ	500 (390–600)	28	-	-
Wild type	19 (16–24)	1	0.6 (0.6–1.0)	1
*hst3*Δ	33 (25–42)	2	1.2 (0.9–1.5)	2
*hst4*Δ	25 (14–41)	1	1.0 (0.5–1.6)	2
*hst3*Δ *hst4*Δ	480 (420–570)	25	14 (11–23)	23
*rtt109*Δ	37 (29–51)	2	5.1 (2.6–7.4)	9
*hst3*Δ *hst4*Δ *rtt109*Δ	30 (19–33)	2	3.1 (2.2–5.2)	5
*H3K56R*	39 (29–56)	2	5.7 (3.4–9.4)	10
*hst3*Δ *hst4*Δ *H3K56R*	32 (28–55)	2	7.0 (3.3–9.6)	12
*H3K56Q*	84^a^ (59–160)	4	5.8^b^ (4.3–9)	10
*hst3*Δ *hst4*Δ *H3K56Q*	120^a^ (83–150)	6	5.0 (3.8–7.3)	8
*hst3*Δ *H3K56Q*	85 (76–140)	5	5.6 (3.9–10)	9
*hst1*Δ *H3K56Q*	96 (77–130)	5	9.3^b^ (5.9–15)	16
*hst1*Δ	19 (16–28)	1	<0.6 (<0.6–0.9)	1
*hst2*Δ	25 (16–32)	1	0.7 (0.4–1.2)	1
*hst1*Δ *hst3*Δ	19 (10–25)	1	1.4 (<0.6–2.2)	2
*hst1*Δ *hst2*Δ *hst3*Δ	19 (15–29)	1	0.5 (0.4–0.7)	1
*hst1*Δ *hst2*Δ *hst4*Δ	15 (11–24)	1	0.8 (0.6–1.0)	1
*hst3*Δ *hst4*Δ *hst1*Δ	1,100^c^ (960–1,500)	58	31 (29–38)	52
*hst3*Δ *hst4*Δ *hst1*Δ *hst2*Δ	1,600^c^ (1,100–1,900)	86	35 (28–53)	58
*hst3*Δ *hst4*Δ *hst2*Δ	680 (440–960)	36	18 (11–32)	30

With the exception of the first four strains, the strains are E134 (wild type) and its mutant derivatives. Fluctuation analyses and calculations of both mutation rates and 95% confidence intervals were performed as described in Materials and Methods. 95% confidence intervals are in parentheses. BY4742 strain lacks the *his7-2* reporter. The difference between two mutation rates marked with

a, b, or c ^a^, ^b^, or ^c^ is not statistically significant (^a^p = 0.17, ^b^p = 0.06, and ^c^p = 0.10).

Hst3 and Hst4 remove acetylations on H3 K56 residues that are introduced by Rtt109 [36]–[40], [46]. No other enzymatic activity has been assigned to Hst3 and Hst4. Based on this information, we hypothesized that Hst3 and Hst4 participate in the suppression of spontaneous mutations (**Table 1**) by deacetylating chromatin histones H3 on K56. If this hypothesis is correct, the loss of H3K56ac by deletion of *RTT109* or introduction of *H3K56R* should make the H3 K56 deacetylation activities of Hst3 and Hst4 unnecessary, and therefore suppress the mutator phenotype of *hst3*Δ *hst4*Δ. (H3K56R variant mimics histone H3 that is not acetylated on lysine 56 [35], [60].) However, if H3 K56 deacetylation by Hst3 and Hst4 is not involved in the protection of yeast genome from spontaneous mutations, the loss of H3K56ac should not affect the mutator phenotype of *hst3*Δ *hst4*Δ. We found that deletion of *RTT109* or introduction of *H3K56R* suppresses the mutator phenotype of *hst3*Δ *hst4*Δ to the level observed in *rtt109*Δ and *H3K56R* (**Table 1**). We concluded from these data that H3 K56 deacetylation by Hst3 and Hst4 is required for the prevention of spontaneous mutations.

H3K56ac is weakly mimicked by H3K56Q [35], [46], [60], [61]. Consistent with H3K56Q being a weak mimic of H3K56ac [46], [60], [61], we observed that the *CAN1* and *his7-2* mutation rates for *H3K56Q* are increased, but 6 and 2 times lower, respectively, than those for *hst3*Δ *hst4*Δ (**Table 1**). Furthermore, we found that the mutation rates for *H3K56Q* and *hst3*Δ *hst4*Δ *H3K56Q* do not differ from each other (**Table 1**). Therefore, these data further support the conclusion that H3 K56 deacetylation by Hst3 and Hst4 is required for mutation avoidance.

Nicotinamide (NAM) is a potent inhibitor of Hst3, Hst4, and other NAD-dependent sirtuins [36], [50], [62]. Yeast strains grown in 25-mM NAM-containing media accumulate an abnormally high level of H3K56ac [36]. We studied whether the presence of NAM in the culture medium promotes spontaneous mutagenesis of several yeast strains. We found that exposure to 25-mM or 50-mM NAM increases the mutation rates in wild type (**Figures 1A and 1B**). For example, the *CAN1* mutation rate for wild type treated with 50-mM NAM increases 30-fold compared to that for untreated wild type. Importantly, our control experiments established that exposing *H3K56R* and *rtt109*Δ to 25-mM or 50-mM NAM has no effect on their mutation rates (**Figures 1A and 1B**). Together, these results provided independent evidence that H3 K56 deacetylation is important for the suppression of spontaneous mutagenesis.

We also found that the *CAN1* and *his7-2* mutation rates in the *hst3*Δ *hst4*Δ strain grown in the presence of 25-mM NAM are twice and five times higher, respectively, than those in untreated strain (**Figures 1A and 1B**). This finding suggested that an NAD-dependent histone deacetylase activity participates in the defense against spontaneous mutations in *hst3*Δ *hst4*Δ.

Hst1 and Hst2 are homologous to Hst3 and Hst4, but their biological functions remain enigmatic [36], [49]. In light of our evidence that the mutation rates in *hst3*Δ *hst4*Δ are increased in the presence of 25-mM NAM (**Figures 1A and 1B**), we sought to determine whether Hst1 and Hst2 are involved in the suppression of spontaneous mutations. We found that the *CAN1* and *his7-2* mutation rates in the *hst1*Δ, *hst2*Δ, *hst1*Δ *hst3*Δ, *hst1*Δ *hst2*Δ *hst3*Δ, and *hst1*Δ *hst2*Δ *hst4*Δ strains are not significantly different from those in wild type (**Table 1**). Furthermore, deletion of *HST2* in the *hst3*Δ *hst4*Δ and *hst3*Δ *hst4*Δ *hst1*Δ strains does not increase spontaneous mutagenesis above the existing levels. However, the mutation rates in *hst3*Δ *hst4*Δ *hst1*Δ are twice higher than those in *hst3*Δ *hst4*Δ (**Table 1**). Together, these findings suggested that Hst1, but not Hst2, contributes to maintaining genome integrity in strains lacking Hst3 and Hst4.

### Spontaneous mutagenesis in strains deficient in H3 K56 acetylation

The histone acetyltransferase Rtt109 produces H3K56ac in the presence of the histone chaperone Asf1 [37]–[40]. We inquired whether H3 K56 acetylation plays a role in mutation avoidance. We determined that deletion of *RTT109* causes 9- and 2-fold increases of the *his7-2* and *CAN1* mutation rates, respectively (**Table 2**). The mutation rates for *asf1*Δ and *H3K56R* are nearly identical to those for *rtt109*Δ (**Table 2**). Importantly, we found that there is epistasis between *H3K56R* and *rtt109*Δ for *CAN1* and *his7-2* mutations (**Table 2**). The simplest interpretation of these results is that H3 K56 acetylation by Rtt109 is involved in a mutation avoidance mechanism that suppresses spontaneous mutations in *his7-2* and *CAN1*.

**Table 2:** Spontaneous mutagenesis in strains deficient in histone H3 K56 acetylation.

	Mutation rate			
	*CAN1*		*his7-2*	
Genotype	Absolute rate (×10^−8^)	Relative rate	Absolute rate (×10^−8^)	Relative rate
Wild type	19 (16–24)	1	0.6 (0.6–1.0)	1
*rtt109*Δ	37 (29–51)	2	5.1 (2.6–7.4)	9
*H3K56R*	39 (29–56)	2	5.7 (3.4–9.4)	10
*asf1*Δ	35 (26–49)	2	8.5 (6.3–10.2)	14
*rtt109*Δ *H3K56R*	44 (34–67)	2	6.7 (3.6–11.7)	11
*htz1*Δ	18 (12–32)	1	0.8 (0.7–1.7)	1
*swr1*Δ	16 (12–24)	1	0.7 (0.5–2.2)	1
*msh2*Δ	620 (450–830)	33	140^a^ (110–170)	230
*msh2*Δ *rtt109*Δ	1,600 (1,000–2,200)	84	230^a^ (170–290)	390
*msh2*Δ *H3K56R*	1,600 (1,000–2,400)	83	510 (350–610)	840
*msh2*Δ *asf1*Δ	1,900 (1,300–3,700)	100	590 (430–920)	990
*pol2-4*	75 (56–100)	4	3.9 (3.5–5)	7
*pol2-4 rtt109*Δ	170 (110–280)	9	16 (9–24)	27
*pol2-4 H3K56R*	370 (240–520)	20	39 (31–56)	65
*pol2-4 asf1*Δ	300 (260–530)	15	19 (17–27)	32
*pol3-5DV*	150 (130–190)	7	9.7 (8–16)	16
*pol3-5DV rtt109*Δ	280 (200–430)	15	20 (15–24)	33
*pol3-5DV H3K56R*	600 (470–920)	32	38 (23–48)	63
*pol3-5DV asf1*Δ	620 (330–800)	32	34 (24–42)	57

a , the two mutation rates are statistically different from each other (p = 0.003).

We considered the possibility that the absence of H3K56ac causes a defect in replication-coupled nucleosome assembly, which in turn increases spontaneous mutagenesis. The current view suggests that normal replication-coupled chromatin assembly in yeast depends on histone chaperones Caf1 (Cac1-Cac2-Cac3 heterotrimer) and Rtt106 [33]. However, as shown in **Table S1**, deletions of the replication histone chaperone genes *CAC2* and *RTT106* have nearly no effect on the *CAN1* and *his7-2* mutation rates. These results suggested that the increased mutagenesis in strains lacking H3K56ac is not caused by defects in the Caf1- and Rtt106-dependent chromatin assembly.

H3K56ac is required for conferring cellular resistance to several DNA-damaging drugs [35]. In this pathway, H3K56ac acts through the ubiquitin ligase containing Rtt101, Mms1, and Mms22 subunits [63], [64]. Our data revealed that deletion of *RTT101*, *MMS1*, or *MMS22* causes an ∼7-fold increase in *his7-2* frameshifts (**Table S2**). Furthermore, we established that *rtt101*Δ and *rtt109*Δ are epistatic for *his7-2* frameshifts (**Table S2**). These results suggested that the Rtt101 cullin-containing ubiquitin ligase is part of an H3 K56 acetylation-dependent mutation avoidance mechanism.

H3K56ac is involved in the regulation of budding yeast transcription [65]. In one mechanism of transcriptional regulation, the presence of H3K56ac leads to the SWR-C-dependent removal of the histone variant H2A.Z from promoter-proximal nucleosomes [65], [66]. In strains deficient in H2A.Z, transcription of ∼320 genes is upregulated while transcription of ∼480 genes is repressed [65]. To test whether the H3K56ac-dependent transcription regulation plays a role in the control of spontaneous mutagenesis, we measured *CAN1* and *his7-2* mutation rates in *htz1*Δ and *swr1*Δ strains. (*HTZ1* is the only gene for the histone variant H2A.Z and *SWR1* encodes the catalytic subunit of the SWR-C chromatin remodeling complex.) As shown in **Table 2**, the *CAN1* and *his7-2* mutation rates in the *htz1*Δ and *swr1*Δ strains are nearly identical to those in wild type. These findings indicated that the defects in the SWR-C- and H2A.Z-dependent transcription regulation do not increase the levels of *can1* and *HIS7* mutations in strains proficient in both the acetylation and deacetylation of H3 K56.

### The deacetylation and acetylation of histone H3 K56 are important for mutation avoidance mechanisms that cooperate with MMR and the proofreading activities of replicative DNA polymerases δ and ε

Analysis of genetic interactions has been critical for understanding the functions of numerous proteins involved in mutation avoidance. Previous studies have defined the existence of multiplicative, synergistic, and additive relationships between mutants that inactivate different mutation avoidance mechanisms [29], [67]–[71]. In a synergistic relationship, the relative mutation rate for a double mutant is greater than the sum of those for the single mutants [29]. A multiplicative relationship is a form of synergistic relationship in which the relative mutation rate in a double mutant is equal to the product of those in the single mutants [29]. The presence of a synergistic or multiplicative relationship indicates that one of the mutants is deficient in one mechanism and the other mutant in a different mechanism, and that the two mechanisms act in concert to suppress the same pool of DNA lesions [29]. On the other hand, the existence of an additive relationship indicates that either mechanism suppresses a different pool of DNA lesions [29]. In an additive relationship, the relative mutation rate for a double mutant is equal to the sum of those for the single mutants [29], [71].

In *hst3*Δ *hst4*Δ strains, nearly all H3 histones are acetylated on K56 at replication forks [36], [47]. We thought that the presence of excess H3K56ac might interfere with high-fidelity DNA replication. Therefore, we decided to test whether histone H3 K56 deacetylation by Hst3 and Hst4 contributes to DNA replication fidelity. In these experiments we used four replication fidelity mutants: *msh2*Δ and *mlh1*Δ completely inactivate MMR [59], [72], *pol2-4* disables the proofreading activity of DNA polymerase ε [73], and *pol3-5DV* eliminates the proofreading activity of DNA polymerase δ [74]. Based on the results of the previous research [29], [67]–[71] described above, we predicted that if a histone H3 K56 deacetylation-dependent mutation avoidance mechanism cooperates with MMR and the proofreading activities of replicative polymerases in promoting replication fidelity, each of triple mutant combinations containing *hst3*Δ *hst4*Δ and one of the replication fidelity mutants (*msh2*Δ, *mlh1*Δ, *pol2-4*, or *pol3-5DV*) should display synergistic or multiplicative, but not additive, increases in the relative *CAN1* and *his7-2* mutation rates. We found that the *hst3*Δ *hst4*Δ *msh2*Δ, *hst3*Δ *hst4*Δ *mlh1*Δ, *hst3*Δ *hst4*Δ *pol2-4*, and *hst3*Δ *hst4*Δ *pol3-5DV* triple mutants indeed show synergistic increases in the relative *CAN1* and *his7-2* mutation rates (**Figure 1C** and **Table S3**). In addition, weak synergies were observed when *hst3*Δ, but not *hst4*Δ, was combined with *msh2*Δ, *mlh1*Δ, *pol2-4*, or *pol3-5DV* (**Table S3**). Taken together, these findings suggested that an H3 K56 deacetylation-dependent mutation avoidance mechanism act in concert with MMR and the proofreading activities of replicative polymerases δ and ε to maintain high-fidelity DNA replication.

Because replication-coupled nucleosome assembly incorporates H3K56ac in chromatin in S phase [35], we tested whether this modification is important for maintaining high-fidelity DNA replication. We found a multiplicative increase in the relative *CAN1* mutation rate when an H3 K56 acetylation mutant (*rtt109*Δ, *H3K56R*, or *asf1*Δ) was combined with a replication fidelity mutant (*msh2*Δ, *pol2-4*, or *pol3-5DV*) (**Table 2**). Furthermore, we established that all these double mutant combinations display synergistic increases in *his7-2* mutation rates (**Table 2**). Collectively, these data suggested that an H3K56ac-dependent mutation avoidance mechanism cooperates with MMR and the proofreading activities of DNA polymerases to promote replication fidelity.

In addition to H3 K56, Rtt109 acetylates other targets [39], [75], [76]. Analysis of data in **Table 2** indicated that the synergies between *rtt109*Δ and replication fidelity mutants for *his7-2* mutations are often weaker than those between *asf1*Δ or *H3K56R* and *msh2*Δ, *pol2-4*, or *pol3-5DV*. Therefore, acetylation of a different target by Rtt109 may compromise replication fidelity.

### Mutations formed in H3 K56 deacetylation- and H3 K56 acetylation-deficient strains

**Figure 2:**
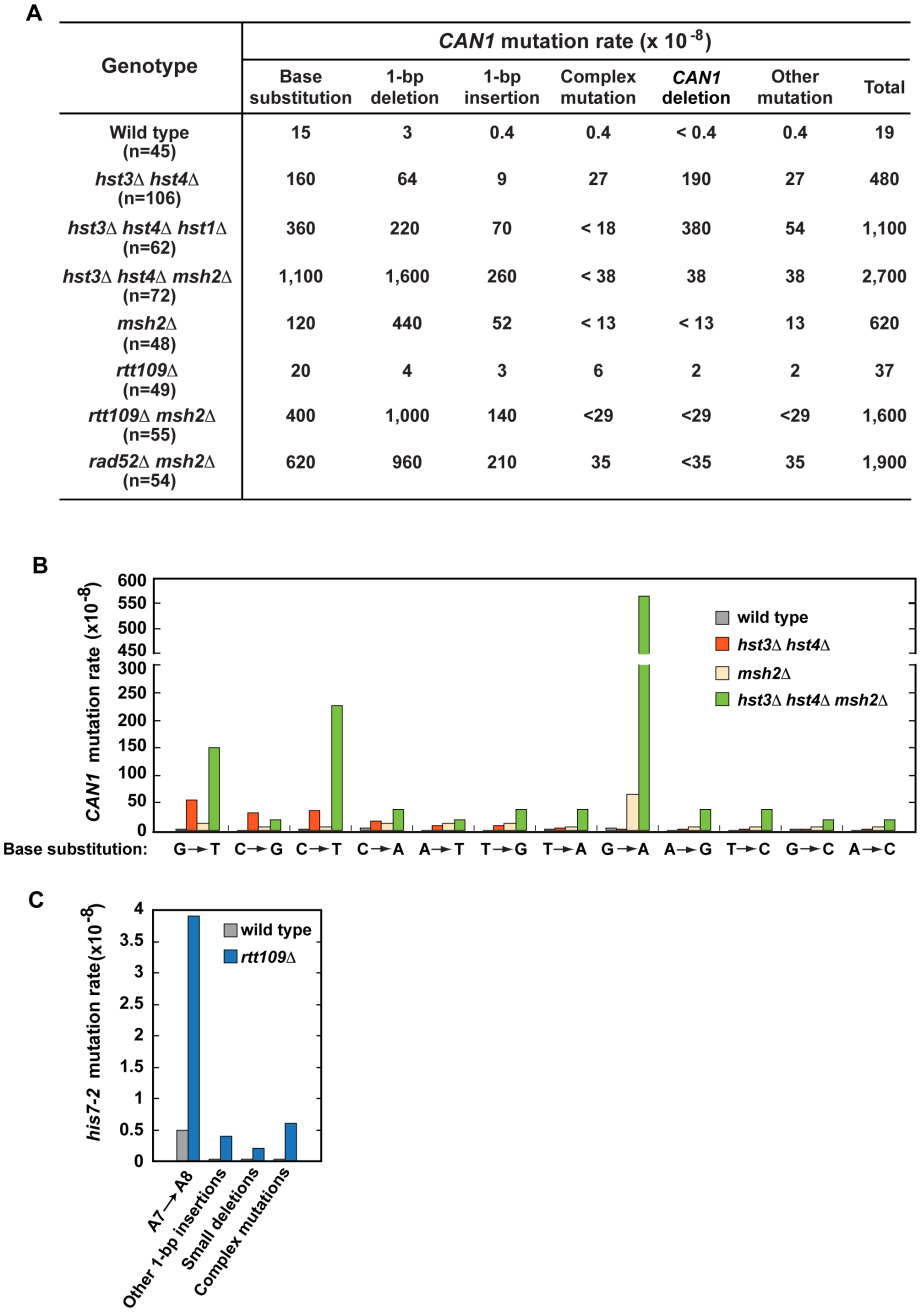
Characterization of spontaneous mutagenesis in *hst3*Δ *hst4*Δ and *rtt109*Δ strains. (**A**) Rates of the different classes of mutations in the coding strand of *CAN1* gene in the indicated strains. The *can1* mutations were identified by DNA sequencing as described in Materials and Methods. Deletions of *CAN1* gene were detected by using PCR reactions like those shown in **Figure S1**. When a genomic DNA did not support PCR amplification of the *CAN1* fragment but produced the *POL2* fragment, the mutant was classified as one that contains a *CAN1* deletion. (**B**) Rates of different *can1* base substitutions in the wild-type and indicated mutant strains. (**C**) Spectra of mutations that reverted *his7-2* in the wild-type and *rtt109*Δ strains. Forty-two mutants of either genotype were sequenced to generate the spectra.

To characterize spontaneous mutagenesis caused by the deficiency in H3 K56 deacetylation (**Figure 1** and **Table 1**), we determined mutations that occurred within the 1.77-kb *CAN1* ORF in the wild-type and *hst3*Δ *hst4*Δ strains by PCRs and DNA sequencing (**Figures 2**, **S1**, **and S2**). Consistent with a previous report [77], we observed that in the wild-type strain 79% of *can1* mutations are base substitutions (**Figure 2A**). Genetic alterations detected in the *hst3*Δ *hst4*Δ strain include base substitutions, 1-bp deletions, 1-bp insertions, complex mutations, and deletions of *CAN1* gene (**Figures 2A** **and S2**). Of those, deletions of *CAN1* gene are the most common mutations generated at a rate of 190×10^−8^. This unexpected finding suggested that strains defective in H3 K56 deacetylaton are very susceptible to GCRs and we confirmed this idea in experiments described in the next subsection. We also found that base substitutions in the *hst3*Δ *hst4*Δ strain accumulate at a high rate of 160×10^−8^. Strikingly, the rate of base substitutions in *hst3*Δ *hst4*Δ is comparable with that in MMR-deficient *msh2*Δ. This finding suggested that H3 K56 deacetylation is a major player in the protection of *S. cerevisiae* from base substitutions. The most common base substitution in the spectrum of *hst3*Δ *hst4*Δ is a G→T transversion produced at a rate of 50×10^−8^ (**Figures 2B** **and S2B**). Analysis of the spectrum also suggested that C→G transversions and C→T transitions are formed at high rates in the H3 K56 deacetylation-deficient strain. Among other mutations detected in *hst3*Δ *hst4*Δ are six medium-size deletions ranging from 40-bp to 1,036-bp. Examination of the end points of the deletions revealed that five out of the six deletions occurred between perfect or nearly perfect direct repeats (**Figure S2D**).

Deletion of *HST1* in *hst3*Δ *hst4*Δ promotes spontaneous mutagenesis (**Table 1**). To obtain additional insight into the interaction between *hst1*Δ and *hst3*Δ *hst4*Δ, we determined *can1* mutation spectrum of *hst3*Δ *hst4*Δ *hst1*Δ. Analysis of the *can1* mutation spectrum showed that the rates of base substitutions, 1-bp deletions, 1-bp insertions, and deletions of *CAN1* gene for *hst3*Δ *hst4*Δ *hst1*Δ are 2–8 times higher than those for *hst3*Δ *hst4*Δ (**Figures 2A**). We also found that the rate of base substitutions for *hst3*Δ *hst4*Δ *hst1*Δ exceeds that for *msh2*Δ by 3-fold. Complex mutations comprising 2 or more mutations within an ∼10-bp DNA are a signature of the action of DNA polymerase ζ [5]. Surprisingly, the mutation spectrum of *hst3*Δ *hst4*Δ *hst1*Δ does not contain even a single complex mutation whereas six complex mutations are present in the spectrum of *hst3*Δ *hst4*Δ (**Figures 2A** **and S2C**). Collectively, these findings suggested that deletion of *HST1* in *hst3*Δ *hst4*Δ significantly affects the dynamics of DNA metabolism.

To characterize the synergy between *hst3*Δ *hst4*Δ and *msh2*Δ (**Figure 1C** and **Table S3**), we determined *can1* mutation spectra of the *msh2*Δ and *hst3*Δ *hst4*Δ *msh2*Δ strains. As expected from the results of an earlier work [72], 71% and 19% of mutations in the *msh2*Δ spectrum are 1-bp deletions and base substitutions, respectively (**Figure 2A**). Analysis of the data indicated that the rate of *CAN1* gene deletions in *hst3*Δ *hst4*Δ *msh2*Δ is 5 times lower than that in *hst3*Δ *hst4*Δ (**Figure 2A**). This result provided us with the first clue that MMR might be involved in the formation of a large fraction of *CAN1* deletions in H3 K56 deacetylation-defective strains. The *can1* mutation spectrum of *hst3*Δ *hst4*Δ *msh2*Δ is dominated by base substitutions and 1-bp deletions accumulating at the rates of 1,100×10^−8^ and 1,600×10^−8^, respectively (**Figure 2A**). Among different base substitutions observed in the spectrum of *hst3*Δ *hst4*Δ *msh2*Δ, G→A changes are the most frequent (**Figures 2B** **and S2B**). Further analysis of the data revealed that there is a synergistic relationship [29] between *hst3*Δ *hst4*Δ and *msh2*Δ for base substitutions, 1-bp deletions, and 1-bp insertions (**Figures S2A and S2B**). For example, the relative rate of G→A substitutions for *hst3*Δ *hst4*Δ *msh2*Δ is 8 times as high as the sum of those for *msh2*Δ and *hst3*Δ *hst4*Δ (**Figure S2B**). Taken together, these findings established that H3 K56 deacetylation cooperates with MMR to prevent base substitutions, 1-bp deletions, and 1-bp insertions.

To better understand the H3 K56 acetylation-dependent suppression of spontaneous mutations, we determined spectra of mutations of the *rtt109*Δ and *rtt109*Δ *msh2*Δ strains (**Figure 2A, 2C** **, and S2A**). Compared to wild type, *rtt109*Δ displays higher rates of 1-bp insertions, complex mutations, and deletions of *CAN1* (**Figure 2A**). The most common mutation in the *his7-2* reporter of the *rtt109*Δ mutant was an A insertion that extended the A_7_ into an A_8_ run (**Figure 2C**). In addition, the *HIS7* spectrum contains other net 1-bp insertions, small deletions, and complex mutations consisting of a 1-bp insertion and an adjacent base substitution. Noticeably, the rate of complex mutations in *his7-2* for *rtt109*Δ is 20 times as high as that for wild type. Collectively, these results demonstrated that H3 K56 acetylation is important for the protection from 1-bp insertions, small deletions, and complex mutations. Comparison of *can1* mutation spectra of the *rtt109*Δ, *msh2*Δ, and *rtt109*Δ *msh2*Δ strains revealed a synergy between *rtt109*Δ and *msh2*Δ for both base substitutions and 1-bp insertions/deletions (**Figure S2A**). Therefore, these data established that an H3 K56 acetylation-dependent mutation avoidance mechanism acts synergistically with MMR to prevent 1-bp deletions, base substitutions, and 1-bp insertions.

### GCRs in *hst3*Δ *hst4*Δ strains

**Figure 3:**
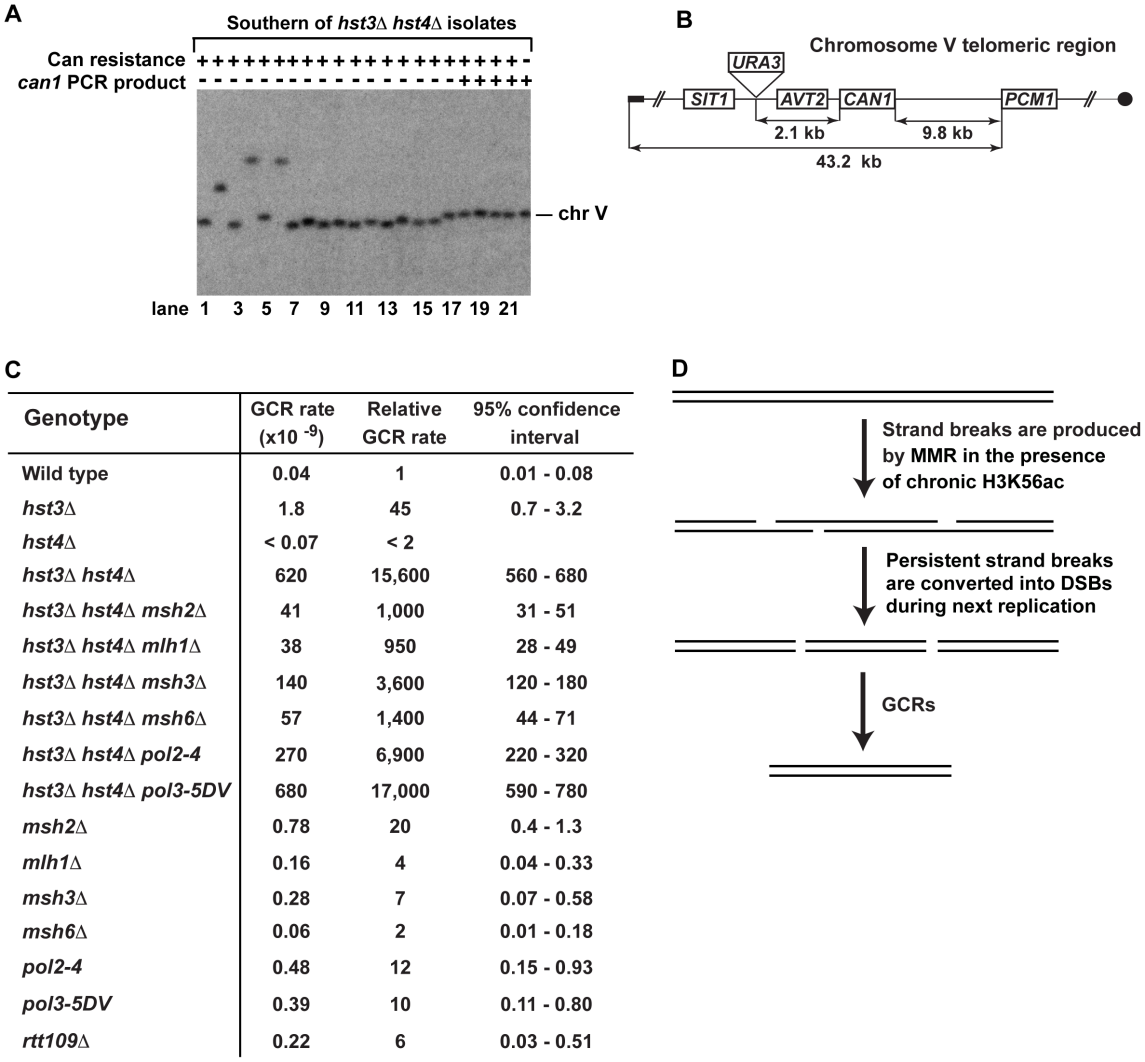
Analysis of GCRs in strains deficient in H3 K56 deacetylation. (**A**) Yeast chromosomes of the indicated genotypes were separated by CHEF gel-electrophoresis, transferred onto a nylon membrane filter, and analyzed by Southern blot hybridization with a ^32^P-labeled probe complementary to a chromosome V region as detailed in Materials and Methods. (**B**) Scheme of the telomeric region of FKY688 strain chromosome V containing *URA3* 2.1-kb telomeric to *CAN1*. (**C**) GCR rates for the FKY688 strain (wild type) and its mutant derivatives. The GCR rates and 95% confidence intervals were determined using the FALCOR web tool [105] as described in Materials and Methods. (**D**) Outline of a possible mechanism that promotes GCRs in *hst3*Δ *hst4*Δ.

The genomic DNAs of 40% of our *can1 hst3*Δ *hst4*Δ isolates did not support PCR amplification of *can1*, but templated the expected *POL2* PCR product (**Figure S1**). This finding implied that the *hst3*Δ *hst4*Δ mutant loses all or part of *CAN1* due to GCRs (**Figure 2A**). To further investigate this phenomenon, we carried out experiments that took advantage of contour-clamped homogenous electric field (CHEF) electrophoresis coupled with Southern blot hybridization. The data revealed the presence of a rearranged chromosome V in *can1 hst3*Δ *hst4*Δ isolates that did not support PCR-based amplification of *can1* (**Figure 3A**). Some of the isolates appear to carry fusions of the chromosome V arm with a different chromosome (**Figure 3A**, lanes 2, 4, and 6), while the other isolates contain deletions within chromosome V (**Figure 3A**, lanes 7–16). Such chromosomal rearrangements have been detected in previous studies [78], [79].

To provide further evidence that the defect in H3 K56 deacetylation triggers GCRs, we measured the rate of GCRs in the wild-type and *hst3*Δ *hst4*Δ strains using an approach developed by Richard Kolodner and coworkers [4]. *URA3* was inserted 2.1-kb telomeric to *CAN1* and the simultaneous loss of the two markers occurring as a result of a GCR was measured (**Figures 3B and 3C**). The rate of GCRs in the *hst3*Δ *hst4*Δ strain is 15,600-fold as high as that in wild type (**Figure 3C**). This finding demonstrated that the lack of H3 K56 deacetylation causes a dramatic increase in the rate of GCRs. Combining *hst3*Δ *hst4*Δ with *pol3-5DV* does not significantly change the rate of GCRs. Strikingly, the *hst3*Δ *hst4*Δ *msh2*Δ and *hst3*Δ *hst4*Δ *mlh1*Δ strains display GCR rates that are 15 times lower than that in isogenic *hst3*Δ *hst4*Δ (**Figure 3C**). Furthermore, we found that deletion of *MSH3* or *MSH6* in the *hst3*Δ *hst4*Δ mutant decreases the rate of GCRs (**Figure 3C**). These results suggested that the formation of the majority of GCRs in *hst3*Δ *hst4*Δ strains involves MMR action dependent on both MutS**α** and MutS**β**. A model shown in **Figure 3D** outlines a possible mechanism of this phenomenon and is described in the Discussion section.

### Analysis of genetic interactions involving H3 K56 deacetylation and H3 K56 acetylation mutants

Next, we investigated whether several DNA repair proteins contribute to the high mutation rates in the *hst3*Δ *hst4*Δ strains (**Table 3**). As described above, the spectrum of *hst3*Δ *hst4*Δ contains complex mutations (**Figures 2A** **and S2C**). Rev3 is the catalytic subunit of DNA polymerase ζ [80] that produces complex and other mutations during replication of damaged and undamaged DNA and during double-strand break repair [71], [81]. The *CAN1* and *his7-2* mutation rates for *rev3*Δ *hst3*Δ *hst4*Δ are nearly identical to those for *hst3*Δ *hst4*Δ. Therefore, these results implied that DNA polymerase ζ is not responsible for the majority of mutations occurring in the H3 K56 deacetylation-deficient strains.

**Table 3:** Involvement of several DNA repair genes in spontaneous mutagenesis in the *hst3*Δ *hst4*Δ and *rtt109*Δ strains.

	Mutation rate			
	*CAN1*		*his7-2*	
Genotype	Absolute rate (×10^−8^)	Relative rate	Absolute rate (×10^−8^)	Relative rate
Wild type	19 (16–24)	1	0.6 (0.6–1.0)	1
*hst3*Δ *hst4*Δ	480 (420–570)	25	14 (11–23)	23
*rev3*Δ	11 (8–17)	0.6	0.6 (<0.6–0.7)	1
*rev3*Δ *hst3*Δ *hst4*Δ	500 (420–830)	26	17 (14–20)	28
*rtt101*Δ	36 (25–71)	2	3.4 (2–7.8)	6
*rtt101*Δ *hst3*Δ *hst4*Δ	41 (36–60)	2	2.3 (1.9–7.3)	4
*ctf18*Δ	25 (19–37)	1	1.7 (0.8–2.8)	3
*ctf18*Δ *hst3*Δ *hst4*Δ	120 (100–180)	6	4.6 (2.6–9.2)	8
*rtt109*Δ	37 (29–51)	2	5.1^a^ (2.6–7.4)	9
*rev3*Δ *rtt109*Δ	18 (16–22)	1	2.6^a^ (1.6–3.1)	4
*rad51*Δ	370 (290–520)	19	4.6 (3.7–5.9)	8
*rad51*Δ *rtt109*Δ	410 (250–430)	22	4.4 (2.8–8.3)	8
*rad52*Δ	390 (310–480)	21	4.0 (3.2–5.7)	7
*rad52*Δ *rtt109*Δ	390 (280–680)	21	5.5 (1.6–8.1)	9

a , the two mutation rates are statistically different from each other (p = 0.013).

Deletion of *RTT101* or *CTF18* suppresses a strong defect of *hst3*Δ *hst4*Δ strains for growth at 37°C [47], [82]. Rtt101 is required for the progression of replication forks through pause sites and damaged DNA template [83], and is part of the H3K56ac-dependent resistance to genotoxic stress [82]. Ctf18 is the largest subunit of the Ctf18-RFC complex, which is essential for sister chromatid cohesion [84] and unloads PCNA from DNA [85]. We analyzed the involvement of both *RTT101* and *CTF18* in the formation of spontaneous mutations in *hst3*Δ *hst4*Δ. As shown in **Table 3**, the *CAN1* and *his7-2* mutation rates in *rtt101*Δ *hst3*Δ *hst4*Δ are 12 and 6 times lower, respectively, than those in *hst3*Δ *hst4*Δ. This finding implicated Rtt101 in the formation of the majority of *can1* and *HIS7* mutations in H3 K56 deacetylation-deficient strains. Furthermore, we found that the mutation rates in *ctf18*Δ *hst3*Δ *hst4*Δ are lower than those in *hst3*Δ *hst4*Δ. This result indicated that Ctf18-RFC is involved in promoting spontaneous mutagenesis in H3 K56 deacetylation-deficient strains.

The presence of complex mutations in the mutation spectra of *rtt109*Δ (**Figures 2A and 2C**) suggested that DNA polymerase ζ might contribute to spontaneous mutagenesis in the H3 K56 acetylation-deficient strains. We determined that deletion of *REV3* in *rtt109*Δ completely suppresses the *CAN1* mutation rate and decreases the *his7-2* mutation rate two-fold (**Table 3**). These results support the idea that DNA polymerase ζ is involved in the formation of mutations in H3 K56 acetylation-deficient strains.

Rad52 and Rad51 are key components of HR [12]. To characterize the genetic interactions between H3 K56 acetylation and HR, we measured the *CAN1* and *his7-2* mutation rates in the *rad51*Δ, *rad51*Δ *rtt109*Δ, *rad52*Δ, and *rad52*Δ *rtt109*Δ strains (**Table 3** **).** Unfortunately, the relative *CAN1* mutation rates in the single and double mutants do not allow us to distinguish between epistasis and additivity in the genetic interactions of *rtt109*Δ with the HR alleles (**Table 3**). However, we found that *rtt109*Δ displays epistatic relationships with *rad51*Δ and *rad52*Δ for *his7-2* mutations (**Table 3**). This finding suggested that the recombination proteins and H3K56 acetylation act in the same pathway to promote the integrity of replication fidelity.

**Figure 4:**
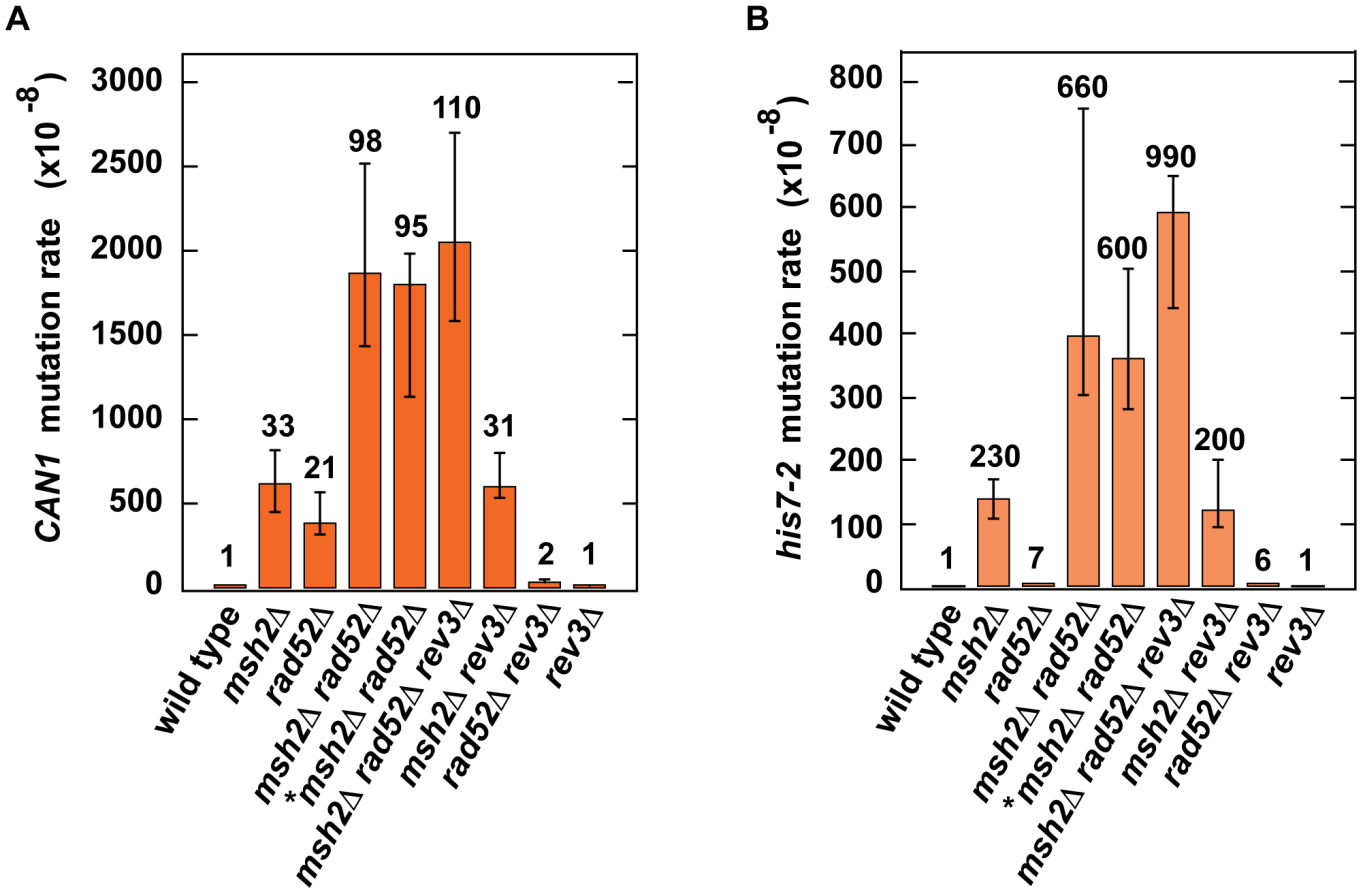
Effect of combining *msh2*Δ with *rad52*Δ on spontaneous mutagenesis. Spontaneous *CAN1* (**A**) and *his7-2* (**B**) mutation rates in the indicated strains are shown. The data are presented as medians with 95% confidence intervals. The relative mutation rates are above the corresponding bars. *, the strain was obtained by tetrad dissection.

Because our results provided evidence that H3K56 acetylation acts synergistically with MMR (**Table 2**) and epistatically with HR (**Table 3**) to control spontaneous mutagenesis, we hypothesized that HR might contribute to fidelity of DNA replication. To test this hypothesis, we studied the genetic interactions between *rad52*Δ and *msh2*Δ (**Figures 4A and 4B**). We found the presence of a synergistic relationship between *rad52*Δ and *msh2*Δ for both *CAN1* and *his7-2* mutations. Rev3 produces the majority of mutations in *rad52* strains by acting on ssDNA generated by the resection of double-strand breaks [86]. We established that in the *rev3*Δ background, the relationship between *rad52*Δ and *msh2*Δ is nearly multiplicative for both *CAN1* and *his7-2* mutations (**Figures 4A and 4B**). Next, we compared the *can1* mutation spectra of the *rad52*Δ *msh2*Δ and *msh2*Δ strains (**Figure 2A**). The mutation spectrum of *rad52*Δ *msh2*Δ is similar to that of *msh2*Δ, but the rates of the most common classes of mutations (1-bp deletions, base substitutions, and 1-bp deletions) for *msh2*Δ are 2–3 times lower than those for *rad52*Δ *msh2*Δ. Taken together, these results suggested that Rad52-dependent HR contributes to fidelity of DNA replication.

## Discussion

**Figure 5:**
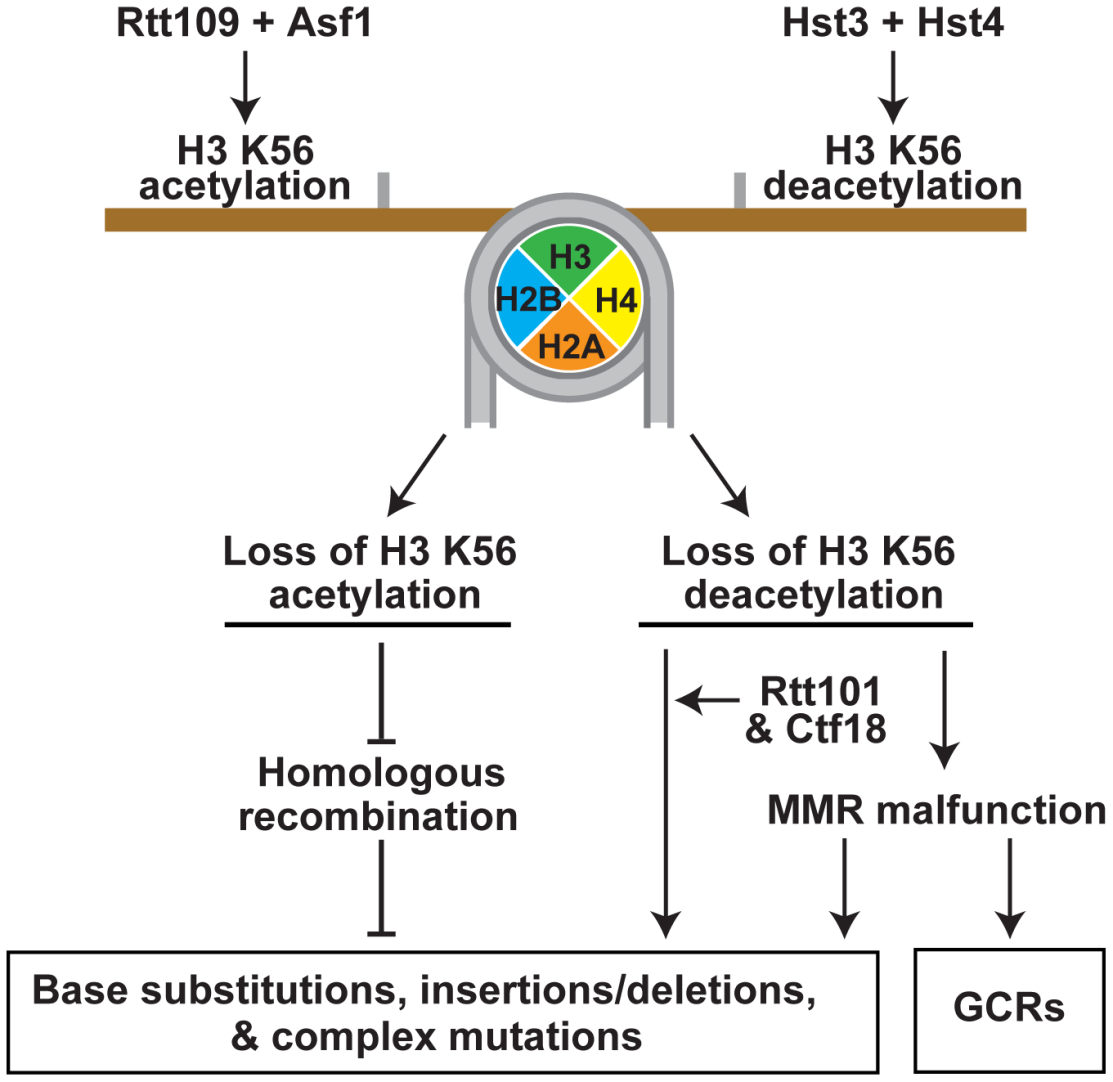
Model that summarizes the importance of the acetylation and deacetylation of H3K56 for the suppression of GCRs, base substitutions, small deletions/insertions, and complex mutations. Rtt109 and Asf1 acetylate newly synthesized histones H3 on K56 prior to their incorporation into chromatin in S phase [37]–[40]. H3K56ac is removed from the new chromatin by Hst3 and Hst4 in G2/M [36], [46], [47]. Hence, in wild-type cells, H3K56ac is present in S phase and G2/M, but absent in G1. In contrast to wild-type cells, *rtt109*Δ and *asf1*Δ lack H3K56ac in S phase and G2/M, and *hst3*Δ *hst4*Δ cells contain H3K56ac in G1. Furthermore, the levels of H3K56ac in S phase and G2/M in *hst3*Δ *hst4*Δ are higher than those in wild type. The imbalance in H3K56ac provides the basis for the different spontaneous mutagenesis in the *rtt109*Δ and *hst3*Δ *hst4*Δ strains.

Chromatin controls many critical aspects of metabolism in eukaryotes. Besides being a major regulator of transcription, chromatin profoundly affects DNA damage response, replication, and repair [32], [33], [87]. However, our knowledge about the relationship between chromatin and spontaneous mutagenesis is very limited. Previous research has identified that yeast Asf1, Caf1, Hst3, and Rtt109-dependent H3 K56 acetylation are involved in the control of GCRs [39], [53]–[55]. Additionally, a recent study reported that SET2D-dependent H3K36me3 regulates the mismatch correction function of human MMR [58]. Up to date, no information has been available about the involvement of either histone acetylation or deacetylation in the protection from point and complex mutations. In yeast *S. cerevisiae*, H3K56ac is an abundant posttranslational modification introduced in and removed from chromatin in a cell cycle-dependent manner [34]–[36], [46]. In this work, we investigated the impact of both the deacetylation and acetylation of H3 K56 on spontaneous mutagenesis in *S. cerevisiae*. We demonstrated that H3 K56 deacetylation by Hst3 and Hst4 plays a critical role in the suppression of GCRs, base substitutions, small insertions/deletions, and complex mutations (**Figures 2**, **3**, and **5**). Remarkably, a strain deficient in Hst3- and Hst4-dependent H3 K56 deacetylation forms GCRs at a rate that is 15,600-fold as high as that in isogenic wild type (**Figure 3C**). Furthermore, we showed that the rates of base substitutions in the *hst3*Δ *hst4*Δ and *msh2*Δ strains are similar to one another (**Figure 2A**). This finding suggests that H3 K56 deacetylation is as important for the prevention of base substitutions as MMR. We also showed that H3 K56 acetylation by Rtt109 and Asf1 is involved in the protection of DNA from 1-bp insertions, small deletions, and complex mutations (**Figures 2A and 2C**); however the effects of H3 K56 acetylation are weaker than those of H3 K56 deacetylation. Therefore, our findings indicate that in addition to controlling gene transcription and GCRs [39], [53]–[55], [87], histone acetylation and deacetylation are required for the defense against point and complex mutations.

This study was greatly facilitated by the availability of the *H3K56R* and *H3K56Q* alleles [35], [46], [60] (**Figure 1** and **Tables 1** and **2**). In our experiments, H3K56R mimicked well H3 unacetylated on K56, but H3K56Q behaved as a weak mimic of H3K56ac. The latter conclusion is based on the observation that the mutation rates for *hst3*Δ *hst4*Δ exceeded those for *H3K56Q* and *hst3*Δ *hst4*Δ *H3K56Q* by 2–6 fold (**Table 1**). Previous studies have also found that H3K56Q mimics weakly H3K56ac [46], [60], [61].

Exposure of the *hst3*Δ *hst4*Δ strain to 25–50-mM NAM increases the mutation rates 2–5-fold (**Figures 1A and 1B** **)**. This finding suggested that another NAD-dependent histone deacetylase is involved in mutation avoidance. Consistent with this, we found that the *CAN1* and *his7-2* mutation rates in *hst1*Δ *hst3*Δ *hst4*Δ are twice higher than those in *hst3*Δ *hst4*Δ (**Table 1**). Given that 8% of histone H3 is still unacetylated on K56 in *hst3*Δ *hst4*Δ strains [36] and that the *CAN1* and *his7-2* mutation rates for *hst1*Δ *H3K56Q* and *H3K56Q* do not differ from each other (**Table 1**), we hypothesize that Hst1 is involved in the suppression of spontaneous mutagenesis in *hst3*Δ *hst4*Δ cells by weakly deacetylating H3 on K56. Alternatively, Hst1 may promote genetic stability by acting on a different target. Since the removal of euchromatic H3K4ac mainly depends on Hst1 [52], H3K4ac may be this target.

Mutagens, in general, produce a lesion by directly acting upon DNA, which is later converted into a mutation. One of the few deviations from this rule is the demonstration that cadmium cations trigger genetic instability in yeast strains by inhibiting an enzymatic system, MMR [88]. We showed that exposure of yeast strains to 50-mM NAM, a specific inhibitor of the NAD-dependent histone deacetylases [62], produces a strong 30-fold increase in the *CAN1* mutation rate (**Figure 1A**). This effect depends on the presence of both H3 K56 and *RTT109*. To the best of our knowledge, these data have provided the first example of a small molecule that inhibits chromatin-modifying enzymes and by doing so strongly promotes spontaneous mutagenesis.

MMR and the proofreading activities of DNA polymerases δ and ε are critical for maintaining high-fidelity DNA replication [2], [18]. We found the presence of synergistic increases in *CAN1* and *his7-2* mutation rates when *hst3*Δ *hst4*Δ is combined with *msh2*Δ, *mlh1*Δ, *pol2-4*, or *pol3-5DV* (**Figures 1C**, **2A, and 2B** and **Table S3**). Furthermore, we established the existence of a synergy between *hst3*Δ *hst4*Δ and *msh2*Δ for base substitutions, 1-bp insertions, and 1-bp deletions (**Figure 2A, 2B** **, and S2A**). It is also evident that the relationships of *rtt109*Δ, *asf1*Δ, and *H3K56R* with *msh2*Δ, *pol2-4*, and *pol3-5DV* are synergistic for *his7-2* frameshifts and multiplicative for *CAN1* mutations (**Table 2**). The presence of synergistic and multiplicative relationships supports the view that both the deacetylation and acetylation of H3 K56 are involved in mutation avoidance pathways that act in concert with MMR and the proofreading activities of the replicative polymerases to promote DNA replication fidelity. The absolute *CAN1* mutation rates for *hst3*Δ *hst4*Δ *msh2*Δ (**Table S3**) and *msh2*Δ *rtt109*Δ (**Table 2**) are 4.2 times and 7.6 times lower, respectively, than that for the *pol2-4 msh2*Δ mutant [69]. Therefore, this comparison suggests that the contributions of the acetylation and deacetylation of H3 K56 to replication fidelity are not as strong as that of the proofreading activity of DNA polymerase ε.

GCRs have been implicated in triggering many different cancers [9]. *S. cerevisiae* has been instrumental for dissecting the mechanisms of GCRs [4], [78], [89], [90]. A study that used a *URA3*-*CAN1* cassette containing the two genes 7.5-kb apart from each other described that deletion of *HST3* or *RTT109* increases the rate of GCRs four-fold [55]. To analyze GCRs, we utilized a *URA3*-*CAN1* cassette in which the distance between *URA3* and *CAN1* is 2.1-kb (**Figures 3B and 3C**). We determined that the deletions of *HST3* and *RTT109* cause 45- and 6-fold increases of the GCR rate, respectively (**Figure 3C**). Surprisingly, the rate of GCRs in *hst3*Δ *hst4*Δ exceeds those in the corresponding single mutants and *rtt109*Δ by at least 350-fold. Therefore, our findings (**Figure 3C**) are in good accord with and extend the previous observations that identified that Hst3 and Rtt109-dependent H3 K56 acetylation play roles in the control of GCRs [39], [53], [89]. In addition, our data (**Figure 3C**) support the view that Hst3 is the principal enzyme for H3 K56 deacetylation [36], [46].

Msh2 and Mlh1 are the key components of yeast MMR [2], [15]. Msh2 is a subunit of the mismatch recognition factors MutSα and MutSβ, whereas Mlh1 forms MutLα endonuclease by dimerizing with Pms1. Strikingly, deletion of *MSH2* or *MLH1* in *hst3*Δ *hst4*Δ reduces the rate of GCRs by 15-fold (**Figure 3C**). On the other hand, the rate of GCRs in the *hst3*Δ *hst4*Δ *pol3-5DV* mutant is nearly identical to that in *hst3*Δ *hst4*Δ. Therefore, these data demonstrate that MMR, but not the proofreading activity of DNA polymerase δ, is required for the generation of the majority of GCRs in the H3 K56 deacetylation-defective strains. We infer from these results that histone H3 K56 deacetylation is necessary to suppress malfunction of MMR. It is possible that in addition to promoting GCRs, MMR malfunction may result in the formation of some point mutations in *hst3*Δ *hst4*Δ (**Figures 2A** and **5**). We speculate that MMR malfunction triggered by a defective environment may be responsible for the formation of a subset of cancer-initiating GCRs and point mutations. It has been known that MMR initiates several neurodegenerative diseases by destabilizing a number of DNA triplet repeats [2]. Thus, the idea that MMR can cause pathogenic consequences has already gained significant experimental support. We also analyzed the importance of the Msh6 subunit of MutSα and the Msh3 subunit of MutSβ for GCR formation (**Figure 3C**). The results of this analysis suggested that both MutSα and MutSβ contribute to the high rate of GCRs in *hst3*Δ *hst4*Δ, but the impact of the latter complex is somewhat weaker compared to that of the former.

How does MMR contribute to the formation of GCRs in *hst3*Δ *hst4*Δ cells? Our data permit us to suggest a speculative model shown in **Figure 3D**. It is known that in the absence of nucleosomes or concomitant nucleosome assembly the MutSα-dependent endonuclease activity of MutLα causes excessive degradation of mismatch-containing DNA in cell-free extracts and defined systems [22], [23], [56]. Therefore, we hypothesize that excessive and persistent nicking of DNA by MutLα may occur in the presence of the defective H3 K56 deacetylation. Such strand breaks can be converted into double-strand breaks in the next round of replication. If DNA flanking an end of one double-strand break carries a sequence that is a direct repeat of DNA flanking an end of another double-strand break, the MutSβ-dependent single strand annealing (SSA) mechanism [91]–[93] can join these two ends producing a GCR. Previous studies already demonstrated the importance of the MutSβ-dependent SSA mechanism for the repair of double-strand breaks flanked by direct repeats [91]–[93]. In this mechanism, MutSβ stabilizes the annealed DNA ends permitting the Rad1-Rad10 nuclease to cleave nonhomologous DNA tails [94]. The involvement of MutSβ and SSA, which is a major mechanism for repairing double-strand breaks flanked by direct repeats [95], in the formation of GCRs in *hst3*Δ *hst4*Δ cells is consistent with the following findings. First, the loss of MutSβ in *hst3*Δ *hst4*Δ decreases the GCR rate four-fold (**Figure 3C**). Second, five out of six identified medium-size deletions in *CAN1* of the *hst3*Δ *hst4*Δ mutant were between direct repeats (**Figure S2D**).

In addition, a different mechanism may lead to GCRs in the *hst3*Δ *hst4*Δ strains. In this mechanism, H3 K56 hyperacetylation, MutSα or MutSβ, and a mismatch activate MutLα endonuclease to initiate the excision of the mismatch on opposite strands. Such aberrant excision may produce a double-strand break. When two double-strand breaks arise in the same *hst3*Δ *hst4*Δ cell, they may be repaired by the SSA mechanism causing a GCR. This mechanism is somewhat related to the one that has been proposed to explain the mismatch repair-dependent killing of *E. coli dam recA* mutants [96].

Though there are strong synergistic relationships between the H3 K56 acetylation mutants (*rtt109*Δ, *asf1*Δ, and *H3K56R*) and the replication fidelity defects (*msh2*Δ, *pol2-4*, and *pol3-5DV*) for *CAN1* mutations, the double mutants show weaker synergistic increases in *his7-2* mutations (**Table 2**). Furthermore, *hst3*Δ *hst4*Δ displays weak synergistic relationships with the MMR-deficient and proofreading mutants for *CAN1* and *his7-2* mutations (**Figure 1C** and **Table S3**). These findings suggest that a large fraction of mutations in both *hst3*Δ *hst4*Δ and *rtt109*Δ strains is produced from DNA lesions/mismatches formed outside S phase. In wild-type strains, H3K56ac appears in S phase and is removed in G2/M [36], [46]. Wild-type, *hst3*Δ, and *hst4*Δ strains do not have H3K56ac in G1, unlike *hst3*Δ *hst4*Δ [36]. Therefore, it is likely that a significant fraction of pre-mutagenic lesions/mismatches in *hst3*Δ *hst4*Δ is formed in G1 as a result of the presence of H3K56ac. Compared to wild type, *rtt109*Δ does not have H3K56ac in S phase and a part of G2/M. Hence, it is possible that S phase-independent pre-mutagenic lesions/mismatches in *rtt109*Δ arise in G2/M as a consequence of the lack of H3K56ac. In addition, the indicated variations in the presence or absence of H3K56ac in the different stages of the cell cycle provide a good explanation of why *hst3*Δ *hst4*Δ and *rtt109*Δ impact spontaneous mutagenesis differently (**Tables 1** **–** **3** and **Figures 2A** and **3C**).

What are the mechanisms that could be responsible for the generation of pre-mutagenic lesions in *hst3*Δ *hst4*Δ during G1 and in *rtt109*Δ during G2/M? Studies of gene transcription have identified many factors that recognize/read the presence or absence of histone modifications including histone acetylations [87], [97]. In addition, some factors read a specific combination of modifications [87]. After forming a complex with a modified/unmodified residue(s), the factor alters transcription of the affected gene. Thus, it is plausible that factors that read the absence or presence of H3K56ac alone or in combination with different modifications in the different stages of the cell cycle change transcription, DNA repair, and/or other mechanisms in a way that results in spontaneous mutagenesis. For example, transcription-dependent variations in the levels of some DNA repair proteins triggered by the defects in the deacetylation or acetylation of H3 K56 may shift the dynamics of DNA metabolism towards increased formation of spontaneous mutations. Consistent with this, it is known that H3K56ac is involved in several mechanisms that regulate gene transcription in yeast [34], [37], [65], and that transcription of ∼370 genes is deregulated in *H3K56Q* cells [65].

One of the mechanisms of transcriptional regulation that involves H3K56ac uses this modification to facilitate SWR-C-dependent removal of the H2A.Z variant from promoter-proximal nucleosomes [65]. Transcription of ∼900 genes is upregulated or downregulated in mutants lacking H2A.Z [65]. We tested whether defects in this H3K56ac-dependent transcriptional regulation affect spontaneous mutagenesis. However, we found that strains lacking H2A.Z or Swr1 do not have increased levels of *CAN1* and *his7-2* mutations (**Table 2**). Thus, the H3K56ac-dependent transcriptional regulation does not contribute to spontaneous mutagenesis in strains with the intact control of both H3 K56 acetylation and H3 K56 deacetylation. Nevertheless, it is still possible that this mechanism of transcriptional regulation contributes to the formation of mutations in strains that are deficient in the acetylation or deacetylation of H3 K56. Furthermore, the defects in the acetylation and deacetylation of H3 K56 may promote the formation of spontaneous mutations via a different mechanism of transcriptional regulation.

The abundant and persistent H3K56ac in *hst3*Δ *hst4*Δ mutants impairs DNA replication [47]. Replication forks in *hst3*Δ *hst4*Δ are able to adapt to the high level of H3K56ac when *RFC1* is overexpressed or *CTF18* is deleted [47]. The overexpression of *RFC1* or deletion of *CTF18* also suppresses the temperature-sensitive phenotype of the cells. Additionally, we observed that deletion of *CTF18* in *hst3*Δ *hst4*Δ strongly reduces the mutation rates (**Table 3**). Because RFC loads PCNA onto DNA and CTF18-RFC unloads the clamp from DNA [85], these results suggest that a higher concentration of PCNA at replication forks allows the cells to adapt to the abundant and persistent H3K56ac. Intriguingly, PCNA is also required for MMR [22], [98], [99]. Thus, we speculate that inadequate concentrations of PCNA at replication forks in *hst3*Δ *hst4*Δ strains may impair both the replicative proofreading and MMR and this compromises replication fidelity. Nevertheless, it is also feasible that factors that recognize unacetylated H3 K56 and promote mutation avoidance cannot be recruited to replication forks in *hst3*Δ *hst4*Δ cells. Alternatively, the presence of the excessive H3K56ac may cause recruitment of mutagenic factors to the replication forks.

What is the mechanism that causes spontaneous mutagenesis in S phase in *rtt109*Δ cells? Our results suggest that H3K56ac is involved in a yet unknown HR mechanism that promotes replication fidelity (**Figure 4** and **Tables 2** and **3**). The progression of replication forks is often impeded by spontaneous DNA damage. Therefore, it is possible that H3K56ac is important for an error-free bypass of spontaneous lesions by the HR machinery during DNA replication. In this mechanism, the presence of H3K56ac may be necessary for recruiting an interacting complex that promotes efficient chromatin remodeling around the lesions and by doing so facilitates an error-free bypass. Understanding the mechanisms that depend on the acetylation and deacetylation of H3 K56 to prevent spontaneous mutagenesis will require further experimentation.

In summary, our findings revealed that the cell cycle-regulated acetylation and deacetylation of chromatin on H3 K56 are critical for suppressing spontaneous mutagenesis (**Figure 5**). The acetylation and deacetylation of H3 K56 are involved in mutation avoidance mechanisms that act in concert with MMR and replicative polymerases to maintain genome stability. The lack of H3K56ac appears to compromise an HR mechanism that promotes replication fidelity. Defective H3 K56 deacetylation causes spontaneous mutagenesis involving Rtt101 and Ctf18, and results in the formation of MMR-dependent GCRs.

## Materials and Methods

### Strains

The *S. cerevisiae* wild-type strains used in this work are E134 (*MAT*α *ade5-1 lys2::InsE-A_14_ trp1-289 his7-2 leu2-3,112 ura3-52*) [68], E35 (*MAT*α *ade5-1 lys2::InsE-A_8_ trp1-289 his7-2 leu2-3,112 ura3-52*) [68], BY4742 (*MAT*α *his3*Δ*1 leu*2Δ*0 lys2*Δ*0 ura3*Δ*0*), and 1B-D770 (*MATa ade5-1 lys2::Tn5-13 trp1-289 his7-2 leu2-3,112 ura3-4*) [59]. Strain SY579 and plasmids pPK588 and pPK589 have previously been described [60], [100]. If not indicated, the mutant strains are derivatives of E134. All strains used in this work are listed in **Table S4**.

To create gene replacements, disruption cassettes with homologous or heterologous markers [101] were amplified in PCRs and introduced into yeast cells by the lithium acetate/PEG-based transformation method [102]. Yeast genomic DNAs were isolated from recombinant isolates with MasterPure Yeast DNA purification kits (Epicentre) and all gene replacements were verified by PCR. The *pol2-4* [67] and *pol3-5DV* [74] alleles were introduced by the integration-excision method. To analyze GCRs, *S. cerevisiae URA3* gene amplified from the pFL34 plasmid with primers (5′- ATACATGCACATATAGCTACTACATAGTCAAGAACATATCATAACATTTGTCTGGCTTTTCAATTCATC-3′) and (5′- GTCGGTAGAGCCAGCATCAGATGCAAAGCCATGCAAAGACTGATATAAAGACTGTTATACAGATCTGAGCTTTTTCTTTCC-3′) was inserted at position 29617 of chromosome V, which is 2,077 bp telomeric to *CAN1* and 3′ adjacent to *SIT1*.

### Measurement of spontaneous mutation rates

Spontaneous mutation rates were measured using fluctuation analysis carried out according to a previously described procedure [59]. On average 15 cultures (no less than 9 cultures), started from single colonies of two-four freshly prepared independent isolates of the same genotype, were used to determine spontaneous mutation rate for this genotype. The cultures were grown to saturation in 3–50 ml YPD medium (1% yeast extract, 2% bacto-peptone, 2% dextrose) supplemented with 60 mg/L adenine and 60 mg/L uracil at 30°C for 20–48 h. When indicated, nicotinamide was added to the supplemented YPD medium to the final concentration of 25 mM or 50 mM. Each saturated culture was plated, after dilution, on a synthetic complete (SC) medium for scoring the total number of cells. The cultures were also plated on SC medium lacking histidine for scoring His^+^ revertants, SC medium lacking arginine and supplemented with 60 mg/L L-canavanine for scoring *can1* mutants, and/or SC medium lacking arginine and supplemented with 60 mg/L L-canavanine and 1 g/L 5-FOA for scoring GCRs. The plates were incubated for 3–5 days at 30°C, and the colonies were counted.

The *CAN1* and *his7-*2 mutation rates were calculated from the total numbers of cells and mutants in the cultures with the Drake formula and are presented as median values with 95% confidence intervals [7], [59]. Where indicated, the significance of observed differences in the *CAN1* and *his7-*2 mutation rates was analyzed with Mann-Whitney U two-tailed test (GraphPad Prism 6 software), where the null hypothesis is that there is no difference between the two data sets. The rates of GCRs were calculated with the Ma-Sandri-Sarkar maximum likelihood method [103], [104] using the web tool FALCOR at http://www.keshavsingh.org/protocols/FALCOR.html [105], [106].

### Analysis of mutation spectra

Mutation spectra in *CAN1* gene were determined essentially as described [71]. Patches were started from single colonies on YPD plates and then replica plated on SC plates supplemented with 60 mg/L L-canavanine and lacking arginine. A single Can^R^ clone from each patch was randomly selected, purified on the selective medium, and then propagated by patching on a YPD plate. Genomic DNAs were isolated from the patched cultures with a MasterPure Yeast DNA purification kit (Epicentre). 2,057-bp fragments containing the entire length of *can1* ORF were amplified with primers 1 (5′-GCAGAAAGAAGAGTGGTTGCGAAC-3′) and 2 (5′-GAGAATGCGAAATGGCGTGGAAATG-3′) in PCR reactions. The amplified fragments were purified with a PCR purification kit (Qiagen) and sequenced such that the entire DNA sequence of the mutant ORF in each clone was determined.

To generate *HIS7* mutation spectra for the wild-type and *rtt109*Δ strains, 1.4-ml or 2.8-ml saturated cultures started from single colonies were concentrated and plated on SC medium lacking histidine. One His^+^ clone from each plate was randomly selected and processed as above. 2005-bp fragments spanning *HIS7* ORF were PCR-amplified with primers 3 (5′-CTCCACGGCTAATTAGGTGATCATG-3′) and 4 (5′-CCTACTGACACCACCAATAATACAAC-3′). The PCR fragments were purified as described above and part of *HIS7* ORF, corresponding to chromosome II coordinates 716234 – 715434, was sequenced. *his7-2* reverts to *HIS7* by acquiring a +1-net frameshift in a 51-bp region (chromosome II coordinates 716023 - 715973) containing an A_7_ run [59].

### CHEF gel electrophoresis and Southern hybridization

Yeast cells were embedded into 0.8% agarose plugs at a concentration of 6×10^8^ cells/ml, and chromosomal DNA was separated by a contour-clamped homogeneous electric field (CHEF) gel electrophoresis in a 1.2% agarose gel/0.5×TBE for 40 hours at 6 V/cm and at 14°C, using the CHEF Mapper XA system (Bio-Rad). The included angle was 120 degrees. The initial and final switch times were 36.63 sec and 2 min 6.67 sec, respectively. The separated yeast chromosomal DNAs were transferred onto a nylon membrane and probed with a ^32^P-labeled *MET6*-specific probe. (The probe was generated by a random prime labeling of a *MET6* PCR fragment amplified with primers 5′-GACGCCATCAAGGGCTTGCCAG-3′ and 5′-CGTTAGCTTCTAGGGCAGCAGC-3′.) The indirectly labeled yeast chromosomal DNAs were visualized with a Kodak BioMax film.

## Supporting Information

Figure S1 PCR analyses of *hst3*Δ *hst4*Δ *can1* mutants. Genomic DNAs of the indicated isolates were prepared as described in Materials and Methods. *can1* mutants were generated in the *hst3*Δ *hst4*Δ (**A**) and *hst3*Δ *hst4*Δ *msh2*Δ (**B**) strains. The PCR analyses of *can1* mutants were carried out with *CAN1* (5′- GCAGAAAGAAGAGTGGTTGCGAAC-3′ and 5′-GAGAATGCGAAATGGCGTGGAAATG-3′) or *POL2* (5′-ATTCCAATCAGTTATTCGAGGCCAG-3′ and 5′-CACCATTGAAGGTGGATATAACAGT-3′) specific primers.
(PDF)

Figure S2 Mutations formed in *hst3*Δ *hst4*Δ and *rtt109*Δ strains. Mutations in the *CAN1* coding strand were identified as detailed in Materials and Methods. (**A**) Relative rates of base substitutions, 1-bp deletions, and 1-bp insertions in the indicated strains. Absolute mutation rates of these classes of mutations are shown in **Figure 2A**. (**B**) Base substitutions in the wild-type, *hst3*Δ *hst4*Δ, *msh2*Δ, and *hst3*Δ *hst4*Δ *msh2*Δ strains. A graphical presentation of these data is shown in **Figure 2B**. The relative rates are in parentheses. (**C**) Complex mutations formed in the *hst3*Δ *hst4*Δ strain. Complex mutations, which are defined as changes of two or more nucleotides within a short segment of DNA [5], are in red. Above of the indicated wild-type sequences of the *CAN1* coding strand (in black) are base substitutions and 1-bp deletions and below are 1-bp and 2-bp insertions. (**D**) Deletions within *CAN1* in the *hst3*Δ *hst4*Δ strain often occurred between direct repeats. PCR and DNA sequencing analyses of *can1* mutants (n = 106) generated in *hst3*Δ *hst4*Δ identified six deletions within *CAN1* ORF. DNA sequences of the 5′ and 3′ junctions of these deletions in the *CAN1* coding strand are shown. Direct repeats that flank a deletion are in red. Parts of the repeats, which were deleted, are underlined.
(PDF)

Table S1 Effect of *CAC2* and *RTT106* deletions on spontaneous mutagenesis.
(DOC)

Table S2 Involvement of several H3 K56 acetylation-dependent DNA damage tolerance genes in the control of spontaneous mutagenesis.
(DOC)

Table S3 Spontaneous mutation rates in strains deficient in H3 K56 deacetylation and replication fidelity.
(DOC)

Table S4 Haploid *S. cerevisiae* strains used in this study.
(DOC)

## References

[1] Kunkel TA (2004) DNA replication fidelity. J Biol Chem 279: 16895–16898. 14988392 10.1074/jbc.R400006200

[2] Iyer RR, Pluciennik A, Burdett V, Modrich PL (2006) DNA mismatch repair: functions and mechanisms. Chem Rev 106: 302–323. 16464007 10.1021/cr0404794

[3] Chen C, Umezu K, Kolodner RD (1998) Chromosomal rearrangements occur in S. cerevisiae rfa1 mutator mutants due to mutagenic lesions processed by double-strand-break repair. Mol Cell 2: 9–22. 9702187 10.1016/s1097-2765(00)80109-4

[4] Chen C, Kolodner RD (1999) Gross chromosomal rearrangements in Saccharomyces cerevisiae replication and recombination defective mutants. Nat Genet 23: 81–85. 10471504 10.1038/12687

[5] Harfe BD, Jinks-Robertson S (2000) DNA polymerase zeta introduces multiple mutations when bypassing spontaneous DNA damage in Saccharomyces cerevisiae. Mol Cell 6: 1491–1499. 11163221 10.1016/s1097-2765(00)00145-3

[6] Kunkel TA, Bebenek K (2000) DNA replication fidelity. Annu Rev Biochem 69: 497–529. 10966467 10.1146/annurev.biochem.69.1.497

[7] Drake JW (1991) A constant rate of spontaneous mutation in DNA-based microbes. Proc Natl Acad Sci USA 88: 7160–7164. 1831267 10.1073/pnas.88.16.7160 PMC52253

[8] Geacintov NE, Swenberg CE (1991) Chemical, molecular biology, and genetic techniques for correlating DNA base damage induced by ionizing radiation with biological end points. Basic Life Sci 58: 453–473 discussion 473–454. 1811481 10.1007/978-1-4684-7627-9_17

[9] Kolodner RD, Putnam CD, Myung K (2002) Maintenance of genome stability in Saccharomyces cerevisiae. Science 297: 552–557. 12142524 10.1126/science.1075277

[10] Malkova A, Haber JE (2012) Mutations arising during repair of chromosome breaks. Annu Rev Genet 46: 455–473. 23146099 10.1146/annurev-genet-110711-155547

[11] Boiteux S, Jinks-Robertson S (2013) DNA Repair Mechanisms and the Bypass of DNA Damage in Saccharomyces cerevisiae. Genetics 193: 1025–1064. 23547164 10.1534/genetics.112.145219 PMC3606085

[12] San Filippo J, Sung P, Klein H (2008) Mechanism of eukaryotic homologous recombination. Annu Rev Biochem 77: 229–257. 18275380 10.1146/annurev.biochem.77.061306.125255

[13] Pursell ZF, Isoz I, Lundstrom EB, Johansson E, Kunkel TA (2007) Yeast DNA polymerase epsilon participates in leading-strand DNA replication. Science 317: 127–130. 17615360 10.1126/science.1144067 PMC2233713

[14] Nick McElhinny SA, Gordenin DA, Stith CM, Burgers PM, Kunkel TA (2008) Division of labor at the eukaryotic replication fork. Mol Cell 30: 137–144. 18439893 10.1016/j.molcel.2008.02.022 PMC2654179

[15] Kolodner RD, Marsischky GT (1999) Eukaryotic DNA mismatch repair. Curr Opin Genet Dev 9: 89–96. 10072354 10.1016/s0959-437x(99)80013-6

[16] Harfe BD, Jinks-Robertson S (2000) DNA Mismatch Repair and Genetic Instability. Annu Rev Genet 34: 359–399. 11092832 10.1146/annurev.genet.34.1.359

[17] Surtees JA, Argueso JL, Alani E (2004) Mismatch repair proteins: key regulators of genetic recombination. Cytogenet Genome Res 107: 146–159. 15467360 10.1159/000080593

[18] Kunkel TA, Erie DA (2005) DNA Mismatch Repair. Annu Rev Biochem 74: 681–710. 15952900 10.1146/annurev.biochem.74.082803.133243

[19] Modrich P (2006) Mechanisms in eukaryotic mismatch repair. J Biol Chem 281: 30305–30309. 16905530 10.1074/jbc.R600022200 PMC2234602

[20] Li GM (2008) Mechanisms and functions of DNA mismatch repair. Cell Res 18: 85–98. 18157157 10.1038/cr.2007.115

[21] Pena-Diaz J, Jiricny J (2012) Mammalian mismatch repair: error-free or error-prone? Trends Biochem Sci 37: 206–214. 22475811 10.1016/j.tibs.2012.03.001

[22] Kadyrov FA, Dzantiev L, Constantin N, Modrich P (2006) Endonucleolytic function of MutLalpha in human mismatch repair. Cell 126: 297–308. 16873062 10.1016/j.cell.2006.05.039

[23] Kadyrov FA, Holmes SF, Arana ME, Lukianova OA, O'Donnell M, et al (2007) Saccharomyces cerevisiae MutLalpha is a mismatch repair endonuclease. J Biol Chem 282: 37181–37190. 17951253 10.1074/jbc.M707617200 PMC2302834

[24] Pluciennik A, Dzantiev L, Iyer RR, Constantin N, Kadyrov FA, et al (2010) PCNA function in the activation and strand direction of MutLalpha endonuclease in mismatch repair. Proc Natl Acad Sci US A 107: 16066–16071. 10.1073/pnas.1010662107 PMC2941292 20713735

[25] Iyer RR, Pluciennik A, Genschel J, Tsai MS, Beese LS, et al (2010) MutLalpha and proliferating cell nuclear antigen share binding sites on MutSbeta. J Biol Chem 285: 11730–11739. 20154325 10.1074/jbc.M110.104125 PMC2857047

[26] Constantin N, Dzantiev L, Kadyrov FA, Modrich P (2005) Human mismatch repair: Reconstitution of a nick-directed bidirectional reaction. J Biol Chem 280: 39752–39761. 16188885 10.1074/jbc.M509701200 PMC1435381

[27] Kadyrov FA, Genschel J, Fang Y, Penland E, Edelmann W, et al (2009) A possible mechanism for exonuclease 1-independent eukaryotic mismatch repair. Proc Natl Acad Sci USA 106: 8495–8500. 19420220 10.1073/pnas.0903654106 PMC2677980

[28] Strand M, Prolla TA, Liskay RM, Petes TD (1993) Destabilization of tracts of simple repetitive DNA in yeast by mutations affecting DNA mismatch repair. Nature 365: 274–276. 8371783 10.1038/365274a0

[29] Morrison A, Johnson AL, Johnston LH, Sugino A (1993) Pathway correcting DNA replication errors in Saccharomyces cerevisiae. EMBO J 12: 1467–1473. 8385605 10.1002/j.1460-2075.1993.tb05790.x PMC413358

[30] Parsons R, Li GM, Longley MJ, Fang WH, Papadopoulos N, et al (1993) Hypermutability and mismatch repair deficiency in RER^+^ tumor cells. Cell 75: 1227–1236. 8261516 10.1016/0092-8674(93)90331-j

[31] Albertson TM, Ogawa M, Bugni JM, Hays LE, Chen Y, et al (2009) DNA polymerase epsilon and delta proofreading suppress discrete mutator and cancer phenotypes in mice. Proc Natl Acad Sci U S A 106: 17101–17104. 19805137 10.1073/pnas.0907147106 PMC2761330

[32] Groth A, Rocha W, Verreault A, Almouzni G (2007) Chromatin challenges during DNA replication and repair. Cell 128: 721–733. 17320509 10.1016/j.cell.2007.01.030

[33] Ransom M, Dennehey BK, Tyler JK (2010) Chaperoning histones during DNA replication and repair. Cell 140: 183–195. 20141833 10.1016/j.cell.2010.01.004 PMC3433953

[34] Xu F, Zhang K, Grunstein M (2005) Acetylation in histone H3 globular domain regulates gene expression in yeast. Cell 121: 375–385. 15882620 10.1016/j.cell.2005.03.011

[35] Masumoto H, Hawke D, Kobayashi R, Verreault A (2005) A role for cell-cycle-regulated histone H3 lysine 56 acetylation in the DNA damage response. Nature 436: 294–298. 16015338 10.1038/nature03714

[36] Celic I, Masumoto H, Griffith WP, Meluh P, Cotter RJ, et al (2006) The sirtuins Hst3 and Hst4p preserve genome integrity by controlling histone h3 lysine 56 deacetylation. Curr Biol 16: 1280–1289. 16815704 10.1016/j.cub.2006.06.023

[37] Schneider J, Bajwa P, Johnson FC, Bhaumik SR, Shilatifard A (2006) Rtt109 is required for proper H3K56 acetylation: a chromatin mark associated with the elongating RNA polymerase II. J Biol Chem 281: 37270–37274. 17046836 10.1074/jbc.C600265200

[38] Han J, Zhou H, Horazdovsky B, Zhang K, Xu RM, et al (2007) Rtt109 acetylates histone H3 lysine 56 and functions in DNA replication. Science 315: 653–655. 17272723 10.1126/science.1133234

[39] Driscoll R, Hudson A, Jackson SP (2007) Yeast Rtt109 promotes genome stability by acetylating histone H3 on lysine 56. Science 315: 649–652. 17272722 10.1126/science.1135862 PMC3334813

[40] Tsubota T, Berndsen CE, Erkmann JA, Smith CL, Yang L, et al (2007) Histone H3-K56 acetylation is catalyzed by histone chaperone-dependent complexes. Mol Cell 25: 703–712. 17320445 10.1016/j.molcel.2007.02.006 PMC1853276

[41] Chen CC, Carson JJ, Feser J, Tamburini B, Zabaronick S, et al (2008) Acetylated lysine 56 on histone H3 drives chromatin assembly after repair and signals for the completion of repair. Cell 134: 231–243. 18662539 10.1016/j.cell.2008.06.035 PMC2610811

[42] Hyland EM, Cosgrove MS, Molina H, Wang D, Pandey A, et al (2005) Insights into the role of histone H3 and histone H4 core modifiable residues in Saccharomyces cerevisiae. Mol Cell Biol 25: 10060–10070. 16260619 10.1128/MCB.25.22.10060-10070.2005 PMC1280264

[43] Franco AA, Lam WM, Burgers PM, Kaufman PD (2005) Histone deposition protein Asf1 maintains DNA replisome integrity and interacts with replication factor C. Genes Dev 19: 1365–1375. 15901673 10.1101/gad.1305005 PMC1142559

[44] Yuan J, Pu M, Zhang Z, Lou Z (2009) Histone H3-K56 acetylation is important for genomic stability in mammals. Cell Cycle 8: 1747–1753. 19411844 10.4161/cc.8.11.8620 PMC2776713

[45] Tjeertes JV, Miller KM, Jackson SP (2009) Screen for DNA-damage-responsive histone modifications identifies H3K9Ac and H3K56Ac in human cells. EMBO J 28: 1878–1889. 19407812 10.1038/emboj.2009.119 PMC2684025

[46] Maas NL, Miller KM, DeFazio LG, Toczyski DP (2006) Cell cycle and checkpoint regulation of histone H3 K56 acetylation by Hst3 and Hst4. Mol Cell 23: 109–119. 16818235 10.1016/j.molcel.2006.06.006

[47] Celic I, Verreault A, Boeke JD (2008) Histone H3 K56 hyperacetylation perturbs replisomes and causes DNA damage. Genetics 179: 1769–1784. 18579506 10.1534/genetics.108.088914 PMC2516057

[48] Munoz-Galvan S, Jimeno S, Rothstein R, Aguilera A (2013) Histone H3K56 acetylation, Rad52, and non-DNA repair factors control double-strand break repair choice with the sister chromatid. PLoS Genet 9: e1003237. 23357952 10.1371/journal.pgen.1003237 PMC3554610

[49] Brachmann CB, Sherman JM, Devine SE, Cameron EE, Pillus L, et al (1995) The SIR2 gene family, conserved from bacteria to humans, functions in silencing, cell cycle progression, and chromosome stability. Genes Dev 9: 2888–2902. 7498786 10.1101/gad.9.23.2888

[50] Hachinohe M, Hanaoka F, Masumoto H (2011) Hst3 and Hst4 histone deacetylases regulate replicative lifespan by preventing genome instability in Saccharomyces cerevisiae. Genes Cells 16: 467–477. 21401809 10.1111/j.1365-2443.2011.01493.x

[51] Haigis MC, Sinclair DA (2010) Mammalian sirtuins: biological insights and disease relevance. Annu Rev Pathol 5: 253–295. 20078221 10.1146/annurev.pathol.4.110807.092250 PMC2866163

[52] Guillemette B, Drogaris P, Lin HH, Armstrong H, Hiragami-Hamada K, et al (2011) H3 lysine 4 is acetylated at active gene promoters and is regulated by H3 lysine 4 methylation. PLoS Genet 7: e1001354. 21483810 10.1371/journal.pgen.1001354 PMC3069113

[53] Myung K, Pennaneach V, Kats ES, Kolodner RD (2003) Saccharomyces cerevisiae chromatin-assembly factors that act during DNA replication function in the maintenance of genome stability. Proc Natl Acad Sci USA 100: 6640–6645. 12750463 10.1073/pnas.1232239100 PMC164500

[54] Chan JE, Kolodner RD (2011) Rapid analysis of Saccharomyces cerevisiae genome rearrangements by multiplex ligation-dependent probe amplification. PLoS Genet 8: e1002539. 22396658 10.1371/journal.pgen.1002539 PMC3291544

[55] Putnam CD, Allen-Soltero SR, Martinez SL, Chan JE, Hayes TK, et al (2012) Bioinformatic identification of genes suppressing genome instability. Proc Natl Acad Sci U S A 109: E3251–3259. 23129647 10.1073/pnas.1216733109 PMC3511103

[56] Kadyrova LY, Rodriges Blanko E, Kadyrov FA (2011) CAF-I-dependent control of degradation of the discontinuous strands during mismatch repair. Proc Natl Acad Sci U S A 108: 2753–2758. 21282622 10.1073/pnas.1015914108 PMC3041128

[57] Schopf B, Bregenhorn S, Quivy JP, Kadyrov FA, Almouzni G, et al (2012) Interplay between mismatch repair and chromatin assembly. Proc Natl Acad Sci U S A 109: 1895–1900. 22232658 10.1073/pnas.1106696109 PMC3277549

[58] Li F, Mao G, Tong D, Huang J, Gu L, et al (2013) The histone mark H3K36me3 regulates human DNA mismatch repair through its interaction with MutSalpha. Cell 153: 590–600. 23622243 10.1016/j.cell.2013.03.025 PMC3641580

[59] Shcherbakova PV, Kunkel TA (1999) Mutator phenotypes conferred by MLH1 overexpression and by heterozygosity for mlh1 mutations. Mol Cell Biol 19: 3177–3183. 10082584 10.1128/mcb.19.4.3177 PMC84111

[60] Recht J, Tsubota T, Tanny JC, Diaz RL, Berger JM, et al (2006) Histone chaperone Asf1 is required for histone H3 lysine 56 acetylation, a modification associated with S phase in mitosis and meiosis. Proc Natl Acad Sci U S A 103: 6988–6993. 16627621 10.1073/pnas.0601676103 PMC1459006

[61] Erkmann JA, Kaufman PD (2009) A negatively charged residue in place of histone H3K56 supports chromatin assembly factor association but not genotoxic stress resistance. DNA Repair (Amst) 8: 1371–1379. 19796999 10.1016/j.dnarep.2009.09.004 PMC2787813

[62] Bitterman KJ, Anderson RM, Cohen HY, Latorre-Esteves M, Sinclair DA (2002) Inhibition of silencing and accelerated aging by nicotinamide, a putative negative regulator of yeast sir2 and human SIRT1. J Biol Chem 277: 45099–45107. 12297502 10.1074/jbc.M205670200

[63] Michel JJ, McCarville JF, Xiong Y (2003) A role for Saccharomyces cerevisiae Cul8 ubiquitin ligase in proper anaphase progression. J Biol Chem 278: 22828–22837. 12676951 10.1074/jbc.M210358200

[64] Krogan NJ, Cagney G, Yu H, Zhong G, Guo X, et al (2006) Global landscape of protein complexes in the yeast Saccharomyces cerevisiae. Nature 440: 637–643. 16554755 10.1038/nature04670

[65] Watanabe S, Radman-Livaja M, Rando OJ, Peterson CL (2013) A histone acetylation switch regulates H2A.Z deposition by the SWR-C remodeling enzyme. Science 340: 195–199. 23580526 10.1126/science.1229758 PMC3727404

[66] Dion MF, Kaplan T, Kim M, Buratowski S, Friedman N, et al (2007) Dynamics of replication-independent histone turnover in budding yeast. Science 315: 1405–1408. 17347438 10.1126/science.1134053

[67] Morrison A, Sugino A (1994) The 3′-->5′ exonucleases of both DNA polymerases delta and epsilon participate in correcting errors of DNA replication in Saccharomyces cerevisiae. Mol Gen Genet 242: 289–296. 8107676 10.1007/BF00280418

[68] Tran HT, Keen JD, Kricker M, Resnick MA, Gordenin DA (1997) Hypermutability of homonucleotide runs in mismatch repair and DNA polymerase proofreading yeast mutants. Mol Cell Biol 17: 2859–2865. 9111358 10.1128/mcb.17.5.2859 PMC232138

[69] Tran HT, Gordenin DA, Resnick MA (1999) The 3′-->5′ exonucleases of DNA polymerases delta and epsilon and the 5′-->3′ exonuclease Exo1 have major roles in postreplication mutation avoidance in Saccharomyces cerevisiae. Mol Cell Biol 19: 2000–2007. 10022887 10.1128/mcb.19.3.2000 PMC83993

[70] Kirchner JM, Tran H, Resnick MA (2000) A DNA polymerase epsilon mutant that specifically causes +1 frameshift mutations within homonucleotide runs in yeast. Genetics 155: 1623–1632. 10924461 10.1093/genetics/155.4.1623 PMC1461198

[71] Northam MR, Robinson HA, Kochenova OV, Shcherbakova PV (2010) Participation of DNA polymerase zeta in replication of undamaged DNA in Saccharomyces cerevisiae. Genetics 184: 27–42. 19841096 10.1534/genetics.109.107482 PMC2815923

[72] Marsischky GT, Filosi N, Kane MF, Kolodner R (1996) Redundancy of *Saccharomyces cerevisiae* MSH3 and MSH6 in MSH2-dependent mismatch repair. Genes Dev 10: 407–420. 8600025 10.1101/gad.10.4.407

[73] Morrison A, Bell JB, Kunkel TA, Sugino A (1991) Eukaryotic DNA polymerase amino acid sequence required for 3′----5′ exonuclease activity. Proc NatlAcad Sci U S A 88: 9473–9477. 10.1073/pnas.88.21.9473 PMC52740 1658784

[74] Jin YH, Obert R, Burgers PM, Kunkel TA, Resnick MA, et al (2001) The 3′-->5′ exonuclease of DNA polymerase delta can substitute for the 5′ flap endonuclease Rad27/Fen1 in processing Okazaki fragments and preventing genome instability. Proc Natl Acad Sci U S A 98: 5122–5127. 11309502 10.1073/pnas.091095198 PMC33174

[75] Fillingham J, Recht J, Silva AC, Suter B, Emili A, et al (2008) Chaperone control of the activity and specificity of the histone H3 acetyltransferase Rtt109. Mol Cell Biol 28: 4342–4353. 18458063 10.1128/MCB.00182-08 PMC2447148

[76] Berndsen CE, Tsubota T, Lindner SE, Lee S, Holton JM, et al (2008) Molecular functions of the histone acetyltransferase chaperone complex Rtt109-Vps75. Nat Struct Mol Biol 15: 948–956. 19172748 10.1038/nsmb.1459 PMC2678805

[77] Kim N, Huang SN, Williams JS, Li YC, Clark AB, et al (2011) Mutagenic processing of ribonucleotides in DNA by yeast topoisomerase I. Science 332: 1561–1564. 21700875 10.1126/science.1205016 PMC3380281

[78] Myung K, Chen C, Kolodner RD (2001) Multiple pathways cooperate in the suppression of genome instability in Saccharomyces cerevisiae. Nature 411: 1073–1076. 11429610 10.1038/35082608

[79] Myung K, Datta A, Kolodner RD (2001) Suppression of spontaneous chromosomal rearrangements by S phase checkpoint functions in *Saccharomyces cerevisiae* . Cell 104: 397–408. 11239397 10.1016/s0092-8674(01)00227-6

[80] Morrison A, Christensen RB, Alley J, Beck AK, Bernstine EG, et al (1989) REV3, a Saccharomyces cerevisiae gene whose function is required for induced mutagenesis, is predicted to encode a nonessential DNA polymerase. J Bacteriol 171: 5659–5667. 2676986 10.1128/jb.171.10.5659-5667.1989 PMC210411

[81] Holbeck SL, Strathern JN (1997) A role for REV3 in mutagenesis during double-strand break repair in Saccharomyces cerevisiae. Genetics 147: 1017–1024. 9383049 10.1093/genetics/147.3.1017 PMC1208230

[82] Collins SR, Miller KM, Maas NL, Roguev A, Fillingham J, et al (2007) Functional dissection of protein complexes involved in yeast chromosome biology using a genetic interaction map. Nature 446: 806–810. 17314980 10.1038/nature05649

[83] Luke B, Versini G, Jaquenoud M, Zaidi IW, Kurz T, et al (2006) The cullin Rtt101p promotes replication fork progression through damaged DNA and natural pause sites. Curr Biol 16: 786–792. 16631586 10.1016/j.cub.2006.02.071

[84] Hanna JS, Kroll ES, Lundblad V, Spencer FA (2001) Saccharomyces cerevisiae CTF18 and CTF4 are required for sister chromatid cohesion. Mol Cell Biol 21: 3144–3158. 11287619 10.1128/MCB.21.9.3144-3158.2001 PMC86942

[85] Bylund GO, Burgers PM (2005) Replication protein A-directed unloading of PCNA by the Ctf18 cohesion establishment complex. Mol Cell Biol 25: 5445–5455. 15964801 10.1128/MCB.25.13.5445-5455.2005 PMC1156988

[86] Roche H, Gietz RD, Kunz BA (1995) Specificities of the Saccharomyces cerevisiae rad6, rad18, and rad52 mutators exhibit different degrees of dependence on the REV3 gene product, a putative nonessential DNA polymerase. Genetics 140: 443–456. 7498727 10.1093/genetics/140.2.443 PMC1206625

[87] Shahbazian MD, Grunstein M (2007) Functions of site-specific histone acetylation and deacetylation. Annu Rev Biochem 76: 75–100. 17362198 10.1146/annurev.biochem.76.052705.162114

[88] Jin YH, Clark AB, Slebos RJ, Al-Refai H, Taylor JA, et al (2003) Cadmium is a mutagen that acts by inhibiting mismatch repair. Nat Genet 34: 326–329. 12796780 10.1038/ng1172 PMC2662193

[89] Putnam CD, Hayes TK, Kolodner RD (2009) Specific pathways prevent duplication-mediated genome rearrangements. Nature 460: 984–989. 19641493 10.1038/nature08217 PMC2785216

[90] Lobachev KS, Gordenin DA, Resnick MA (2002) The Mre11 complex is required for repair of hairpin-capped double-strand breaks and prevention of chromosome rearrangements. Cell 108: 183–193. 11832209 10.1016/s0092-8674(02)00614-1

[91] Saparbaev M, Prakash L, Prakash S (1996) Requirement of mismatch repair genes MSH2 and MSH3 in the RAD1-RAD10 pathway of mitotic recombination in Saccharomyces cerevisiae. Genetics 142: 727–736. 8849883 10.1093/genetics/142.3.727 PMC1207014

[92] Sugawara N, Paques F, Colaiacovo M, Haber JE (1997) Role of *Saccharomyces cerevisiae* Msh2 and Msh3 repair proteins in double-strand break-induced recombination. Proc Natl Acad Sci U S A 94: 9214–9219. 9256462 10.1073/pnas.94.17.9214 PMC23120

[93] Studamire B, Price G, Sugawara N, Haber JE, Alani E (1999) Separation-of-function mutations in Saccharomyces cerevisiae MSH2 that confer mismatch repair defects but do not affect nonhomologous-tail removal during recombination. Mol Cell Biol 19: 7558–7567. 10523644 10.1128/mcb.19.11.7558 PMC84769

[94] Lyndaker AM, Alani E (2009) A tale of tails: insights into the coordination of 3′ end processing during homologous recombination. Bioessays 31: 315–321. 19260026 10.1002/bies.200800195 PMC2958051

[95] Haber JE (1995) In vivo biochemistry: physical monitoring of recombination induced by site-specific endonucleases. Bioessays 17: 609–620. 7646483 10.1002/bies.950170707

[96] McGraw BR, Marinus MG (1980) Isolation and characterization of *dam* ^+^ revertants and suppressor mutations that modify secondary phenotypes of *dam-3* strains of *Escherichia coli* K-12. Mol Gen Genet 178: 309–315. 6993844 10.1007/BF00270477

[97] Yun M, Wu J, Workman JL, Li B (2011) Readers of histone modifications. Cell Res 21: 564–578. 21423274 10.1038/cr.2011.42 PMC3131977

[98] Umar A, Buermeyer AB, Simon JA, Thomas DC, Clark AB, et al (1996) Requirement for PCNA in DNA mismatch repair at a step preceding DNA resynthesis. Cell 87: 65–73. 8858149 10.1016/s0092-8674(00)81323-9

[99] Dzantiev L, Constantin N, Genschel J, Iyer RR, Burgers PM, et al (2004) A defined human system that supports bidirectional mismatch-provoked excision. Mol Cell 15: 31–41. 15225546 10.1016/j.molcel.2004.06.016

[100] Sutton A, Bucaria J, Osley MA, Sternglanz R (2001) Yeast ASF1 protein is required for cell cycle regulation of histone gene transcription. Genetics 158: 587–596. 11404324 10.1093/genetics/158.2.587 PMC1461693

[101] Gueldener U, Heinisch J, Koehler GJ, Voss D, Hegemann JH (2002) A second set of loxP marker cassettes for Cre-mediated multiple gene knockouts in budding yeast. Nucleic Acids Res 30: e23. 11884642 10.1093/nar/30.6.e23 PMC101367

[102] Gietz RD, Woods RA (2002) Transformation of yeast by lithium acetate/single-stranded carrier DNA/polyethylene glycol method. Methods Enzymol 350: 87–96. 12073338 10.1016/s0076-6879(02)50957-5

[103] Ma WT, GV Sandri GV, Sarkar S (1992) Analysis of the Luria-Delbrück distribution using discrete convolution powers. J Appl Prob 29: 255–267.

[104] Asteris G, Sarkar S (1996) Bayesian procedures for the estimation of mutation rates from fluctuation experiments. Genetics 142: 313–326. 8770608 10.1093/genetics/142.1.313 PMC1206960

[105] Hall BM, Ma CX, Liang P, Singh KK (2009) Fluctuation analysis CalculatOR: a web tool for the determination of mutation rate using Luria-Delbruck fluctuation analysis. Bioinformatics 25: 1564–1565. 19369502 10.1093/bioinformatics/btp253 PMC2687991

[106] Rosche WA, Foster PL (2000) Determining mutation rates in bacterial populations. Methods 20: 4–17. 10610800 10.1006/meth.1999.0901 PMC2932672

