# Supplementary figures and images for: A Reversible Histone H3 Acetylation Cooperates with Mismatch Repair and Replicative Polymerases in Maintaining Genome Stability
Source: PLoS Genet. 2013 Oct 24;9(10):e1003899. doi: 10.1371/journal.pgen.1003899 (PMC3812082; doi:10.1371/journal.pgen.1003899)

**A**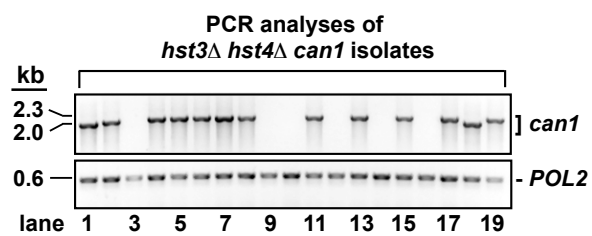**B**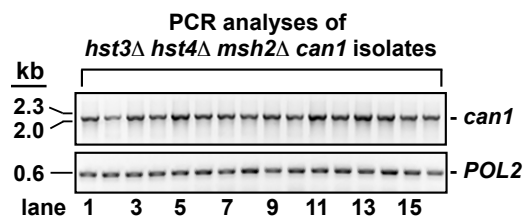

Supplement: Figure S1 — PCR analyses of hst3Δ hst4Δ can1 mutants. Genomic DNAs of the indicated isolates were prepared as described in Materials and Methods. can1 mutants were generated in the hst3Δ hst4Δ (A) and hst3Δ hst4Δ msh2Δ (B) strains. The PCR analyses of can1 mutants were carried out with CAN1 (5′- GCAGAAAGAAGAGTGGTTGCGAAC-3′ and 5′-GAGAATGCGAAATGGCGTGGAAATG-3′) or POL2 (5′-ATTCCAATCAGTTATTCGAGGCCAG-3′ and 5′-CACCATTGAAGGTGGATATAACAGT-3′) specific primers. (PDF) [file pgen.1003899.s001.pdf]
