# Supplementary material for: A Reversible Histone H3 Acetylation Cooperates with Mismatch Repair and Replicative Polymerases in Maintaining Genome Stability
Source: PLoS Genet. 2013 Oct 24;9(10):e1003899. doi: 10.1371/journal.pgen.1003899 (PMC3812082; doi:10.1371/journal.pgen.1003899)
Supplement: Figure S2 — Mutations formed in hst3Δ hst4Δ and rtt109Δ strains. Mutations in the CAN1 coding strand were identified as detailed in Materials and Methods. (A) Relative rates of base substitutions, 1-bp deletions, and 1-bp insertions in the indicated strains. Absolute mutation rates of these classes of mutations are shown in Figure 2A . (B) Base substitutions in the wild-type, hst3Δ hst4Δ, msh2Δ, and hst3Δ hst4Δ msh2Δ strains. A graphical presentation of these data is shown in Figure 2B . The relative rates are in parentheses. (C) Complex mutations formed in the hst3Δ hst4Δ strain. Complex mutations, which are defined as changes of two or more nucleotides within a short segment of DNA [5], are in red. Above of the indicated wild-type sequences of the CAN1 coding strand (in black) are base substitutions and 1-bp deletions and below are 1-bp and 2-bp insertions. (D) Deletions within CAN1 in the hst3Δ hst4Δ strain often occurred between direct repeats. PCR and DNA sequencing analyses of can1 mutants (n = 106) generated in hst3Δ hst4Δ identified six deletions within CAN1 ORF. DNA sequences of the 5′ and 3′ junctions of these deletions in the CAN1 coding strand are shown. Direct repeats that flank a deletion are in red. Parts of the repeats, which were deleted, are underlined. (PDF) [file pgen.1003899.s002.pdf]

A

Genotype	Relative *CAN1* mutation rates		
	Base substitution	1-bp deletion	1-bp insertion
Wild type (n=45)	1	1	1
hst3Δ hst4Δ (n=106)	11	21	23
hst3Δ hst4Δ hst1Δ (n=62)	24	73	180
hst3Δ hst4Δ msh2Δ (n=72)	73	530	650
msh2Δ (n=48)	8	150	130
rtt109Δ (n=49)	1	1	8
rtt109Δ msh2Δ (n=55)	27	330	350
rad52Δ msh2Δ (n=54)	41	320	530

B

Genotype	Base substitution mutation rates (x 10^{-8})											
	G→A	C→T	G→T	C→A	A→T	T→G	T→A	C→G	A→G	T→C	G→C	A→C
Wild type	3.4 (1)	1.3	1.3	4.2	< 0.4	< 0.4	1.3	0.4	< 0.4	< 0.4	1.3	0.8
hst3Δ hst4Δ	< 5 (< 2)	32	50	18	9	9	5	32	< 5	< 5	< 5	< 5
msh2Δ	65 (19)	< 13	13	13	13	13	< 13	< 13	< 13	< 13	< 13	< 13
hst3Δ hst4Δ msh2Δ	566 (167)	227	151	38	< 38	38	38	< 38	38	38	< 38	< 38

C

Chromosome V coordinates	Complex *can1* mutations in *hst3Δ hst4Δ*
33,310 - 33,299	G<br>5′- CGCTCTTTCCCG -3′<br>CC
33,178 - 33,170	T C<br>5′- GCCCTTGGT -3′
32,933 - 32,924	AΔ G<br>5′- AATACTTTT -3′<br>A
32,581 - 32,573	A T<br>5′- ACATTTCAA -3′
32,352 - 32,342	G<br>5′- AGGTTTTGCCA -3′<br>TT
32,010 - 32,005	TΔ<br>5′- ACGAGT -3′

D

Chromosome V coordinates	Deletion size (bp)	Nucleotide sequences at the deletion junctions
32,922 - 32,883	40	5′-TGGGCAATCACTTTTG.....CAATTTTGGACGTACA-3′
32,879 - 32,359	521	5′-TTTTGGACGTACAAAG.....GTACAAAGGTTTTGCC-3′
33,461 - 32,426	1,036	5′-AGGCATAGCAATGAC.....CATACAATGACCCTAA-3′
32,969 - 32,166	804	5′-TTTCTCCAGCATTTGG.....GCATTTGGCGCTTTGG-3′
32,931 - 32,836	96	5′-GGTTTTCTTGGGCAAT.....TTTTTTGGGTAATTATCA-3′
31,847 - 31,706	142	5′-CCTGTTCTTAGCTGTT.....TTGTAGCATAGATATGAC-3′
