# Supplementary material for: A Reversible Histone H3 Acetylation Cooperates with Mismatch Repair and Replicative Polymerases in Maintaining Genome Stability
Source: PLoS Genet. 2013 Oct 24;9(10):e1003899. doi: 10.1371/journal.pgen.1003899 (PMC3812082; doi:10.1371/journal.pgen.1003899)
Supplement: Table S1 — Effect of CAC2 and RTT106 deletions on spontaneous mutagenesis. (DOC) [file pgen.1003899.s003.doc]

Table S1.

|  | **Mutation rate** | | | |
| --- | --- | --- | --- | --- |
|  | ***CAN1*** | | ***his7-2*** | |
| **Genotype** | **Absolute rate (x10^-8^)** | **Relative rate** | **Absolute rate (x10^-8^)** | **Relative rate** |
| **Wild type** | **19 (16 – 24)** | **1** | **0.6 (0.6 – 1.0)** | **1** |
| ***cac2*∆** | **29 (15 – 39)** | **1.5** | **0.7 (0.4 – 1.1)** | **1** |
| ***rtt106*∆** | **17 (8 – 27)** | **1** | **0.9 (< 0.6 – 1.3)** | **1.5** |
| ***cac2*∆ *rtt106*∆** | **19 (12 – 37)** | **1** | **1.1 (< 1.3 – 2.3)** | **2** |

Fluctuation tests and calculations of mutation rates and 95% confidence intervals were performed as described in Materials and Methods. 95% confidence intervals are in parentheses.
