# Supplementary material for: A Reversible Histone H3 Acetylation Cooperates with Mismatch Repair and Replicative Polymerases in Maintaining Genome Stability
Source: PLoS Genet. 2013 Oct 24;9(10):e1003899. doi: 10.1371/journal.pgen.1003899 (PMC3812082; doi:10.1371/journal.pgen.1003899)
Supplement: Table S2 — Involvement of several H3 K56 acetylation-dependent DNA damage tolerance genes in the control of spontaneous mutagenesis. (DOC) [file pgen.1003899.s004.doc]

**Table S2.**

|  | **Mutation rate** | |
| --- | --- | --- |
|  | ***his7-2*** | |
| **Genotype** | **Absolute rate (x10^-8^)** | **Relative rate** |
| **Wild type** | **0.6 (0.4 – 1.0)** | **1** |
| ***rtt101*∆** | **3.8 (2.9 – 4.2)** | **6** |
| ***rtt109∆*** | **4.4 (3.6 – 10.5)** | **7** |
| ***rtt101∆ rtt109∆*** | **4.9 (3.4 – 6.1)** | **8** |
| ***mms1∆*** | **4.4 (3.3 – 5.8)** | **7** |
| ***mms22∆*** | **4.2 (3.7 – 5.7)** | **7** |

The analyzed strains are E35 (wild type) and its mutant derivatives.
