# Supplementary material for: A Reversible Histone H3 Acetylation Cooperates with Mismatch Repair and Replicative Polymerases in Maintaining Genome Stability
Source: PLoS Genet. 2013 Oct 24;9(10):e1003899. doi: 10.1371/journal.pgen.1003899 (PMC3812082; doi:10.1371/journal.pgen.1003899)
Supplement: Table S3 — Spontaneous mutation rates in strains deficient in H3 K56 deacetylation and replication fidelity. (DOC) [file pgen.1003899.s005.doc]

Table S3.

|  | **Mutation rate** | | | |
| --- | --- | --- | --- | --- |
|  | ***CAN1*** | | ***his7-2*** | |
| **Genotype** | **Absolute rate (x10^-8^)** | **Relative rate** | **Absolute rate (x10^-8^)** | **Relative rate** |
| **Wild type** | **19 (16 – 24)** | **1** | **0.6 (0.6 – 1.0)** | **1** |
| ***hst3∆*** | **33 (25 – 42)** | **2** | **1.2 (0.9 – 1.5)** | **2** |
| ***hst4∆*** | **25 (14 – 41)** | **1** | **1.0 (0.5 – 1.6)** | **2** |
| ***hst3∆ hst4∆*** | **480 (420 – 570)** | **25** | **14 (11 – 23)** | **23** |
| ***H3K56Q*** | **84 (59 – 160)** | **4** | **5.8 (4.3 – 9)** | **10** |
| ***msh2∆*** | **620 ^a, b^ (450 – 830)** | **33** | **140 ^c, d^ (110 – 170)** | **230** |
| ***hst3∆ msh2∆*** | **990 ^a^ (730 – 1,460)** | **52** | **200 ^c^ (160 – 260)** | **340** |
| ***hst4∆ msh2∆*** | **710 ^b^ (490 – 850)** | **37** | **140 ^d^ (130 – 180)** | **240** |
| ***hst3∆ hst4∆ msh2∆*** | **2,700 (1,500 – 3,880)** | **140** | **520 (310 – 700)** | **870** |
| ***H3K56Q msh2∆*** | **2,300 (1,700 – 2,800)** | **120** | **530 (390 – 620)** | **890** |
| ***mlh1∆*** | **500 ^e, f^ (340 – 800)** | **26** | **120 ^g, h^ (78 – 170)** | **200** |
| ***hst3∆ mlh1∆*** | **840 ^e^ (660 – 1,100)** | **44** | **210 ^g^ (190 – 230)** | **350** |
| ***hst4∆ mlh1∆*** | **500 ^f^ (450 – 620)** | **24** | **120 ^h^ (100 – 150)** | **200** |
| ***hst3∆ hst4∆ mlh1∆*** | **2,600 (1,900 – 3,400)** | **130** | **590 (300 – 750)** | **990** |
| ***pol2-4*** | **75 (56 – 100)** | **4** | **3.9 (3.5 – 5)** | **7** |
| ***hst3∆ pol2-4*** | **99 (83 – 140)** | **5** | **10 (4.5 – 15)** | **17** |
| ***hst4∆ pol2-4*** | **63 (51 – 97)** | **3** | **5.8 (4.3– 9.4)** | **10** |
| ***hst3∆ hst4∆ pol2-4*** | **1,100 (850 – 1,700)** | **58** | **98 (80 – 160)** | **160** |
| ***pol3-5DV*** | **150 (130 – 190)** | **7** | **9.7 (8 – 16)** | **16** |
| ***hst3∆ pol3-5DV*** | **340 (240 – 540)** | **18** | **12 (8 – 21)** | **20** |
| ***hst4∆ pol3-5DV*** | **230 (160 – 250)** | **12** | **8.8 (5.8 – 13)** | **15** |
| ***hst3∆ hst4∆ pol3-5DV*** | **3,400 (2,000 – 4,300)** | **180** | **69 (37 – 100)** | **120** |

The difference between two mutation rates marked with ^a^, ^c^, ^e^, or ^g^ is statistically significant (^a^p=0.015, ^c^p=0.005, ^e^p=0.016 and ^g^p=0.0001), whereas the difference between two mutation rates marked with ^b^, ^d^, ^f^, or ^h^ is not (^b^p=0.53, ^d^p=0.67, ^f^p=0.85 and ^h^p=0.71).
