# Supplementary material for: A Reversible Histone H3 Acetylation Cooperates with Mismatch Repair and Replicative Polymerases in Maintaining Genome Stability
Source: PLoS Genet. 2013 Oct 24;9(10):e1003899. doi: 10.1371/journal.pgen.1003899 (PMC3812082; doi:10.1371/journal.pgen.1003899)
Supplement: Table S4 — Haploid S. cerevisiae strains used in this study. (DOC) [file pgen.1003899.s006.doc]

**Table S4.**

| **Strain** | **Genotype** |
| --- | --- |
| **E134 and its derivatives** | |
| **E134** | *MAT*α *ade5-1 lys2::InsE-A_14_ trp1-289 his7-2 leu2-3,112 ura3-52* |
| **FKY1** | *MAT*α *ade5-1 lys2::InsE-A_14_ trp1-289 his7-2 leu2-3,112 ura3-52* *cac2:TRP1* |
| **FKY8** | *MAT*α *ade5-1 lys2::InsE-A_14_ trp1-289 his7-2 leu2-3,112 ura3-52* *ctf18::KanMX* |
| **FKY17** | *MAT*α *ade5-1 lys2::InsE-A_14_ trp1-289 his7-2 leu2-3,112 ura3-52* *cac2:TRP1 rtt106::KanMX* |
| **FKY49** | *MAT*α *ade5-1 lys2::InsE-A_14_ trp1-289 his7-2 leu2-3,112 ura3-52* *msh2::LEU2* |
| **FKY52** | *MAT*α *ade5-1 lys2::InsE-A_14_ trp1-289 his7-2 leu2-3,112 ura3-52* *asf1::URA3 msh2::LEU2* |
| **FKY56** | *MAT*α *ade5-1 lys2::InsE-A_14_ trp1-289 his7-2 leu2-3,112 ura3-52* *asf1::URA3* |
| **FKY62** | *MAT*α *ade5-1 lys2::InsE-A_14_ trp1-289 his7-2 leu2-3,112 ura3-52* *rtt109::LEU2* |
| **FKY87** | *MAT*α *ade5-1 lys2::InsE-A_14_ trp1-289 his7-2 leu2-3,112 ura3-52* *pol2-4* |
| **FKY97** | *MAT*α *ade5-1 lys2::InsE-A_14_ trp1-289 his7-2 leu2-3,112 ura3-52* *pol2-4 asf1::URA3* |
| **FKY106** | *MAT*α *ade5-1 lys2::InsE-A_14_ trp1-289 his7-2 leu2-3,112 ura3-52* *pol3-5DV* |
| **FKY115** | *MAT*α *ade5-1 lys2::InsE-A_14_ trp1-289 his7-2 leu2-3,112 ura3-52* *pol3-5DV asf1::URA3* |
| **FKY132** | *MAT*α *ade5-1 lys2::InsE-A_14_ trp1-289 his7-2 leu2-3,112 ura3-52* *rev3::LEU2* |
| **FKY184** | *MAT*α *ade5-1 lys2::InsE-A_14_ trp1-289 his7-2 leu2-3,112 ura3-52* *rtt109::LEU2 msh2::URA3* |
| **FKY188** | *MAT*α *ade5-1 lys2::InsE-A_14_ trp1-289 his7-2 leu2-3,112 ura3-52* *rtt106::KanMX* |
| **FKY211** | *MAT*α *ade5-1 lys2::InsE-A_14_ trp1-289 his7-2 leu2-3,112 ura3-52* *pol2-4 rtt109::LEU2* |
| **FKY244** | *MAT*α *ade5-1 lys2::InsE-A_14_ trp1-289 his7-2 leu2-3,112 ura3-52* *hht2-hhf2::URA3 hht1-hhf1::LEU2 pPK588*[*TRP1 CEN HHT2(K56Q)-HHF2*] |
| **FKY257** | *MAT*α *ade5-1 lys2::InsE-A_14_ trp1-289 his7-2 leu2-3,112 ura3-52* *hst3::LEU2* |
| **FKY263** | *MAT*α *ade5-1 lys2::InsE-A_14_ trp1-289 his7-2 leu2-3,112 ura3-52* *pol3-5DV rtt109::URA3* |
| **FKY273** | *MAT*α *ade5-1 lys2::InsE-A_14_ trp1-289 his7-2 leu2-3,112 ura3-52* *rev3::LEU2 rtt109::URA3* |
| **FKY275** | *MAT*α *ade5-1 lys2::InsE-A_14_ trp1-289 his7-2 leu2-3,112 ura3-52* *hht2-hhf2::URA3 hht1-hhf1::LEU2 msh2::KanMX pPK588*[*TRP1 CEN HHT2(K56Q)-HHF2*] |
| **FKY278** | *MAT*α *ade5-1 lys2::InsE-A_14_ trp1-289 his7-2 leu2-3,112 ura3-52* *hst3::LEU2 hst4::KanMX* |
| **FKY292** | *MAT*α *ade5-1 lys2::InsE-A_14_ trp1-289 his7-2 leu2-3,112 ura3-52* *hht2-hhf2::URA3 hht1-hhf1::LEU2 pPK589*[*TRP1 CEN HHT2(K56R)-HHF2*] |
| **FKY293** | *MAT*α *ade5-1 lys2::InsE-A_14_ trp1-289 his7-2 leu2-3,112 ura3-52* *hst4::KanMX* |
| **FKY309** | *MAT*α *ade5-1 lys2::InsE-A_14_ trp1-289 his7-2 leu2-3,112 ura3-52* *mlh1::KanMX* |
| **FKY371** | *MAT*α *ade5-1 lys2::InsE-A_14_ trp1-289 his7-2 leu2-3,112 ura3-52* *hst3::LEU2 hst4::KanMX rtt109::URA3* |
| **FKY374** | *MAT*α *ade5-1 lys2::InsE-A_14_ trp1-289 his7-2 leu2-3,112 ura3-52* *hst3::LEU2 hst4::KanMX msh2::URA3* |
| **FKY376** | *MAT*α *ade5-1 lys2::InsE-A_14_ trp1-289 his7-2 leu2-3,112 ura3-52* *rad52::KanMX* |
| **FKY387** | *MAT*α *ade5-1 lys2::InsE-A_14_ trp1-289 his7-2 leu2-3,112 ura3-52* *pol3-5DV hst3::LEU2* |
| **FKY390** | *MAT*α *ade5-1 lys2::InsE-A_14_ trp1-289 his7-2 leu2-3,112 ura3-52* *pol2-4 hst3::LEU2* |
| **FKY393** | *MAT*α *ade5-1 lys2::InsE-A_14_ trp1-289 his7-2 leu2-3,112 ura3-52* *pol2-4 hht2-hhf2::URA3 hht1-hhf1::LEU2 pPK589*[*TRP1 CEN HHT2(K56R)-HHF2*] |
| **FKY400** | *MAT*α *ade5-1 lys2::InsE-A_14_ trp1-289 his7-2 leu2-3,112 ura3-52* *pol3-5DV hht2-hhf2::URA3 hht1-hhf1::LEU2 pPK589*[*TRP1 CEN HHT2(K56R)-HHF2*] |
| **FKY402** | *MAT*α *ade5-1 lys2::InsE-A_14_ trp1-289 his7-2 leu2-3,112 ura3-52* *pol2-4 hst3::LEU2 hst4::KanMX* |
| **FKY405** | *MAT*α *ade5-1 lys2::InsE-A_14_ trp1-289 his7-2 leu2-3,112 ura3-52* *pol3-5DV hst3::LEU2 hst4::KanMX* |
| **FKY409** | *MAT*α *ade5-1 lys2::InsE-A_14_ trp1-289 his7-2 leu2-3,112 ura3-52* *hht2-hhf2::URA3 hht1-hhf1::LEU2 msh2::KanMX pPK589*[*TRP1 CEN HHT2(K56R)-HHF2*] |
| **FKY412** | *MAT*α *ade5-1 lys2::InsE-A_14_ trp1-289 his7-2 leu2-3,112 ura3-52* *hst3::LEU2 hst4::KanMX rtt101::URA3* |
| **FKY416** | *MAT*α *ade5-1 lys2::InsE-A14 trp1-289 his7-2 leu2-3,112 ura3-52* *rtt109::LEU2 rad52::KanMX* |
| **FKY419** | *MAT*α *ade5- lys2::InsE-A_14_ trp1-289 his7-2 leu2-3,112 ura3-52* *rad51::URA3* |
| **FKY447** | *MAT*α *ade5-1 lys2::InsE-A_14_ trp1-289 his7-2 leu2-3,112 ura3-52* *hst3::LEU2 hst4::KanMX rev3::URA3* |
| **FKY475** | *MAT*α *ade5-1 lys2::InsE-A_14_ trp1-289 his7-2 leu2-3,112 ura3-52* *msh2::LEU2 rad52::KanMX* |
| **FKY501** | *MAT*α *ade5-1 lys2::InsE-A_14_ trp1-289 his7-2 leu2-3,112 ura3-52* *rtt109::LEU2 rad51::URA3* |
| **FKY516** | *MAT*α *ade5-1 lys2::InsE-A_14_ trp1-289 his7-2 leu2-3,112 ura3-52* *rev3::LEU2 rad52::URA3* |
| **FKY525** | *MAT*α *ade5-1 lys2::InsE-A_14_ trp1-289 his7-2 leu2-3,112 ura3-52* *rev3::LEU2 msh2::URA3* |
| **FKY562** | *MAT*α *ade5-1 lys2::InsE-A_14_ trp1-289 his7-2 leu2-3,112 ura3-52* *rev3::LEU2 msh2::URA3 rad52::KanMX* |
| **FKY599** | *MAT*α *ade5-1 lys2::InsE-A_14_ trp1-289 his7-2 leu2-3,112 ura3-52* *hst3::LEU2 hst4::KanMX hst1::URA3* |
| **FKY611** | *MAT*α *ade5-1 lys2::InsE-A_14_ trp1-289 his7-2 leu2-3,112 ura3-52* *hst3::LEU2 hst4::KanMX ctf18::URA3* |
| **FKY626** | *MAT*α *ade5-1 lys2::InsE-A_14_ trp1-289 his7-2 leu2-3,112 ura3-52* *hst3::LEU2 hst4::KanMX mlh1::URA3* |
| **FKY633** | *MAT*α *ade5-1 lys2::InsE-A_14_ trp1-289 his7-2 leu2-3,112 ura3-52* *hst3::LEU2 msh2::URA3* |
| **FKY636** | *MAT*α *ade5-1 lys2::InsE-A_14_ trp1-289 his7-2 leu2-3,112 ura3-52* *hst4::KanMX msh2::URA3* |
| **FKY642** | *MAT*α *ade5-1 lys2::InsE-A_14_ trp1-289 his7-2 leu2-3,112 ura3-52* *hht2-hhf2::URA3 hht1-hhf1::LEU2 rtt109::KanMX pPK589*[*TRP1 CEN HHT2(K56R)-HHF2*] |
| **FKY646** | *MAT*α *ade5-1 lys2::InsE-A_14_ trp1-289 his7-2 leu2-3,112 ura3-52* *pol2-4 hst4::KanMX* |
| **FKY649** | *MAT*α *ade5-1 lys2::InsE-A_14_ trp1-289 his7-2 leu2-3,112 ura3-52* *pol3-5DV hst4::KanMX* |
| **FKY652** | *MAT*α *ade5-1 lys2::InsE-A_14_ trp1-289 his7-2 leu2-3,112 ura3-52* *hht2-hhf2::URA3 hht1-hhf1::LEU2 hst3::HphMX hst4::KanMX pPK589*[*TRP1 CEN HHT2(K56R)-HHF2*] |
| **FKY655** | *MAT*α *ade5-1 lys2::InsE-A_14_ trp1-289 his7-2 leu2-3,112 ura3-52* *hst4::KanMX mlh1::URA3* |
| **FKY659** | *MAT*α *ade5-1 lys2::InsE-A_14_ trp1-289 his7-2 leu2-3,112 ura3-52* *hst3::LEU2 mlh1::URA3* |
| **FKY679** | *MAT*α *ade5-1 lys2::InsE-A_14_ trp1-289 his7-2 leu2-3,112 ura3-52* *hht2-hhf2::URA3 hht1-hhf1::LEU2 hst1::KanMX pPK588*[*TRP1 CEN HHT2(K56Q)-HHF2*] |
| **FKY682** | *MAT*α *ade5-1 lys2::InsE-A_14_ trp1-289 his7-2 leu2-3,112 ura3-52* *hht2-hhf2::URA3 hht1-hhf1::LEU2 hst3::HphMX pPK588*[*TRP1 CEN HHT2(K56Q)-HHF2*] |
| **FKY685** | *MAT*α *ade5-1 lys2::InsE-A_14_ trp1-289 his7-2 leu2-3,112 ura3-52* *hst3::LEU2 hst1::URA3* |
| **FKY688** | *MAT*α *ade5-1 lys2::InsE-A_14_ trp1-289 his7-2 leu2-3,112 ura3-52* *V29617::URA3* |
| **FKY695** | *MAT*α *ade5-1 lys2::InsE-A_14_ trp1-289 his7-2 leu2-3,112 ura3-52* *V29617::URA3 mlh1::KanMX* |
| **FKY698** | *MAT*α *ade5-1 lys2::InsE-A_14_ trp1-289 his7-2 leu2-3,112 ura3-52* *V29617::URA3 msh2::LEU2* |
| **FKY703** | *MAT*α *ade5-1 lys2::InsE-A_14_ trp1-289 his7-2 leu2-3,112 ura3-52* *V29617::URA3 pol2-4* |
| **FKY710** | *MAT*α *ade5-1 lys2::InsE-A_14_ trp1-289 his7-2 leu2-3,112 ura3-52* *V29617::URA3 pol3-5DV* |
| **FKY758** | *MAT*α *ade5-1 lys2::InsE-A_14_ trp1-289 his7-2 leu2-3,112 ura3-52* *V29617::URA3 pol2-4 hst3::HphMX hst4::KanMX* |
| **FKY760** | *MAT*α *ade5-1 lys2::InsE-A_14_ trp1-289 his7-2 leu2-3,112 ura3-52 V29617::URA3* *pol3-5DV hst3::HphMX hst4::KanMX* |
| **FKY767** | *MAT*α *ade5-1 lys2::InsE-A_14_ trp1-289 his7-2 leu2-3,112 ura3-52* *V29617::URA3 rtt109::LEU2* |
| **FKY771** | *MAT*α *ade5-1 lys2::InsE-A_14_ trp1-289 his7-2 leu2-3,112 ura3-52* *V29617::URA3 hst4::KanMX* |
| **FKY778** | *MAT*α *ade5-1 lys2::InsE-A_14_ trp1-289 his7-2 leu2-3,112 ura3-52* *V29617::URA3 hst4::KanMX hst3::LEU2* |
| **FKY796** | *MAT*α *ade5-1 lys2::InsE-A_14_ trp1-289 his7-2 leu2-3,112 ura3-52* *V29617::URA3 hst4::KanMX hst3::LEU2 mlh1::HphMX* |
| **FKY800** | *MAT*α *ade5-1 lys2::InsE-A_14_ trp1-289 his7-2 leu2-3,112 ura3-52* *V29617::URA3 hst4::KanMX hst3::LEU2 msh2::HphMX* |
| **FKY804** | *MAT*α *ade5-1 lys2::InsE-A_14_ trp1-289 his7-2 leu2-3,112 ura3-52* *rtt101::URA3* |
| **FKY808** | *MAT*α *ade5-1 lys2::InsE-A_14_ trp1-289 his7-2 leu2-3,112 ura3-52* *hst2::URA3* |
| **FKY812** | *MAT*α *ade5-1 lys2::InsE-A_14_ trp1-289 his7-2 leu2-3,112 ura3-52* *hst3::LEU2 hst4:KanMX* *hst2::URA3* |
| **FKY816** | *MAT*α *ade5-1 lys2::InsE-A_14_ trp1-289 his7-2 leu2-3,112 ura3-52* *V29617::URA3 hst3::HphMX* |
| **FKY820** | *MAT*α *ade5-1 lys2::InsE-A_14_ trp1-289 his7-2 leu2-3,112 ura3-52* *hst1::URA3* |
| **FKY879** | *MAT*α *ade5-1 lys2::InsE-A_14_ trp1-289 his7-2 leu2-3,112 ura3-52 hht2-hhf2::URA3 hht1-hhf1::LEU2 hst3::HphMX hst4::KanMX pPK588*[*TRP1 CEN HHT2(K56Q)-HHF2*] |
| **FKY882** | *MAT*α *ade5-1 lys2::InsE-A_14_ trp1-289 his7-2 leu2-3,112 ura3-52* *V29617::URA3 hst4::KanMX hst3::LEU2 msh3::HphMX* |
| **FKY885** | *MAT*α *ade5-1 lys2::InsE-A_14_ trp1-289 his7-2 leu2-3,112 ura3-52* *V29617::URA3 hst4::KanMX hst3::LEU2 msh6::HphMX* |
| **FKY888** | *MAT*α *ade5-1 lys2::InsE-A_14_ trp1-289 his7-2 leu2-3,112 ura3-52* *V29617::URA3 msh3::HphMX* |
| **FKY891** | *MAT*α *ade5-1 lys2::InsE-A_14_ trp1-289 his7-2 leu2-3,112 ura3-52* *V29617::URA3 msh6::HphMX* |
| **FKY894** | *MAT*α *ade5-1 lys2::InsE-A_14_ trp1-289 his7-2 leu2-3,112 ura3-52 htz1::HphMX* |
| **FKY897** | *MAT*α *ade5-1 lys2::InsE-A_14_ trp1-289 his7-2 leu2-3,112 ura3-52 hst1::URA3 hst2::HphMX hst3::LEU2* |
| **FKY900** | *MAT*α *ade5-1 lys2::InsE-A_14_ trp1-289 his7-2 leu2-3,112 ura3-52 hst1::URA3 hst2::HphMX hst4::KanMX* |
| **FKY903** | *MAT*α *ade5-1 lys2::InsE-A_14_ trp1-289 his7-2 leu2-3,112 ura3-52 swr1::HphMX* |
| **FKY908** | *MAT*α *ade5-1 lys2::InsE-A_14_ trp1-289 his7-2 leu2-3,112 ura3-52 hst1::URA3 hst2::HphMX hst4::KanMX hst3::LEU2* |
| **E35 and its derivatives** | |
| **E35** | *MAT*α *ade5-1 lys2::InsE-A_8_ trp1-289 his7-2 leu2-3,112 ura3-52* |
| **FKY397** | *MAT*α *ade5-1 lys2::InsE-A_8_ trp1-289 his7-2 leu2-3,112 ura3-52* *rtt101::URA3* |
| **FKY428** | *MAT*α *ade5-1 lys2::InsE-A_8_ trp1-289 his7-2 leu2-3,112 ura3-52* *rtt101::URA3 rtt109::LEU2* |
| **FKY456** | *MAT*α *ade5-1 lys2::InsE-A_8_ trp1-289 his7-2 leu2-3,112 ura3-52* *mms22::URA3* |
| **FKY459** | *MAT*α *ade5-1 lys2::InsE-A_8_ trp1-289 his7-2 leu2-3,112 ura3-52* *rtt109::LEU2* |
| **FKY478** | *MAT*α *ade5-1 lys2::InsE-A_8_ trp1-289 his7-2 leu2-3,112 ura3-52* *hst3::LEU2 hst4::KanMX* |
| **FKY489** | *MAT*α *ade5-1 lys2::InsE-A_8_ trp1-289 his7-2 leu2-3,112 ura3-52* *mms1::URA3* |
| **BY4742 and its derivative** | |
| **BY4742** | *MAT*α *his3*∆*1 leu2*∆*0 lys2*∆*0 ura3*∆*0* |
| **FKY751** | *MAT*α *his3*∆*1 leu2*∆*0 lys2*∆*0 ura3*∆*0 hst3::HphMX hst4::KanMX* |
| **1B-D770 and its derivative** | |
| **1B-D770** | *MATa ade5-1 lys2::Tn5-13 trp1-289 his7-2 leu2-3,112 ura3-4* |
| **FKY439** | *MATa ade5-1 lys2::Tn5-13 trp1-289 his7-2 leu2-3,112 ura3-4 msh2::URA3* |
| **A derivative of FKY376 and FKY439** | |
| **FKY490^a^** | *MAT*α *ade5-1 lys2::InsE-A_14_ trp1-289 his7-2 leu2-3,112 msh2::URA3 rad52::KanMX* |
| **A derivative of W303** | |
| **SY579** | *MAT*α *asf1::URA3 cac2::TRP1* |

^a^ Strain FKY490 was obtained by sporulation and tetrad dissection of a diploid strain derived from a cross of FKY376 and FKY439.
